# Cell-type specificity of ChIP-predicted transcription factor binding sites

**DOI:** 10.1186/1471-2164-13-372

**Published:** 2012-08-03

**Authors:** Tony Håndstad, Morten Rye, Rok Močnik, Finn Drabløs, Pål Sætrom

**Affiliations:** 1 Department of Cancer Research and Molecular Medicine, Norwegian University of Science and Technology, Trondheim, NO-7491, Norway; 2 Department of Computer and Information Science, Norwegian University of Science and Technology, Trondheim, NO-7491, Norway

## Abstract

**Background:**

Context-dependent transcription factor (TF) binding is one reason for differences in gene expression patterns between different cellular states. Chromatin immunoprecipitation followed by high-throughput sequencing (ChIP-seq) identifies genome-wide TF binding sites for one particular context—the cells used in the experiment. But can such ChIP-seq data predict TF binding in other cellular contexts and is it possible to distinguish context-dependent from ubiquitous TF binding?

**Results:**

We compared ChIP-seq data on TF binding for multiple TFs in two different cell types and found that on average only a third of ChIP-seq peak regions are common to both cell types. Expectedly, common peaks occur more frequently in certain genomic contexts, such as CpG-rich promoters, whereas chromatin differences characterize cell-type specific TF binding. We also find, however, that genotype differences between the cell types can explain differences in binding. Moreover, ChIP-seq signal intensity and peak clustering are the strongest predictors of common peaks. Compared with strong peaks located in regions containing peaks for multiple transcription factors, weak and isolated peaks are less common between the cell types and are less associated with data that indicate regulatory activity.

**Conclusions:**

Together, the results suggest that experimental noise is prevalent among weak peaks, whereas strong and clustered peaks represent high-confidence binding events that often occur in other cellular contexts. Nevertheless, 30-40% of the strongest and most clustered peaks show context-dependent regulation. We show that by combining signal intensity with additional data—ranging from context independent information such as binding site conservation and position weight matrix scores to context dependent chromatin structure—we can predict whether a ChIP-seq peak is likely to be present in other cellular contexts.

## Background

Transcription factors (TFs) are proteins that bind sequence elements in DNA and thereby affect expression of neighboring or distal genes. Depending on cellular contexts, such as hormone stimulus or the cell’s differentiation state or cell type, a TF can bind to different subsets of the TF’s potential binding sites and regulate different gene expression programs [1]. Investigating this context-dependent binding of TFs and the causes of binding differences across different cellular contexts is therefore fundamental for understanding gene regulation in general, and also for understanding how differential binding by TFs contribute to disease development.

There are three main factors that determine a TF’s binding activity at a potential binding site. First, TFs bind to specific sequence motifs [2] that favor a local DNA structure recognized by the TF’s DNA-binding domain. Second, the local chromatin structure needs to be favorable for TF binding. Specifically, the chromatin must be sufficiently accessible to allow the TF to scan and bind to its sequence motif [3-5]—a process that is influenced both by high level chromatin structure and local nucleosome positioning [5,6]. Certain post-translational histone modifications are associated with open or closed chromatin and therefore also binding site activity, but certain TFs may also directly bind specific histone modifications [7-9]. Similarly, DNA methylation also affects TF binding—both by directly affecting binding motifs and by being involved in altering local chromatin structure [10]. Third, TF co-activators can recruit and stabilize TF binding, whereas repressors can out-compete or hinder binding to a potential binding site [11].

The TF binding activities that result from a given cellular context form in sum a transcription regulatory network. There are many different methods of inferring the structure of such regulatory networks *in silico*. Some of these methods rely on context-dependent data, such as experimentally determined gene expression, TF binding, or chromatin structure [12], and therefore produce networks specific to a given context. Examples include methods that rely on gene expression data only [13,14], and methods that integrate expression data and binding location data [15-18].

In comparison, many traditional methods for inferring regulatory networks are context-indifferent, typically relying on sequence motifs to map putative transcription factor binding sites (TFBS). Some of these methods use additional data such as a putative site’s conservation level in related species [19,20] and motif clustering [21-24] to increase the predictions’ signal to noise ratio [25]. However, newer methods increasingly take advantage of recently available experimental data such as genome-wide occurrences of histone modifications and nucleosome occupancy [26,27] and our increased understanding of how these modifications affect the likelihood of TF binding [28]. Unlike previous methods that mainly rely on sequence motifs, adding experimental data typically makes the predictions specific to the given experimental context.

Chromatin immunoprecipitation followed by massively parallel DNA sequencing (ChIP-seq) is the current high-throughput experimental technique of choice for mapping the genome-wide state of chromatin, and this technique is also used for experimentally identifying TFBS [12]. ChIP-seq captures TF binding as it happens *in vivo*, so using ChIP-seq data alone or as a basis in more integrative methods for modeling gene regulation will result in context-specific predictions [17,18]. But how specific are these predictions to the given context?

The few studies that have investigated cell-type specificity of TFBS show that in general, binding differences increase with functional and evolutionary distance. A study investigating MyoD-binding in the highly related cell types myoblasts and myotubes found the majority of predicted binding sites to be common in both tissues [29]. Another study looking at E2F4 binding sites in seven primary mouse tissues and a mouse cell line found that between 65% and 85% of the cells’ binding events overlapped [30], whereas a study of serum response factor (SRF) binding across three distinct human cell lines found that less than half of the observed SRF binding sites were shared across all three cell lines [31]. Studies comparing TFBS across homologous species have shown that TFBS in general are even less conserved between different species than between different cells within the same organism [30,32,33]. Thus, whether regulatory interactions determined for one cellular context can be used to predict functional outcomes in a different context seems to depend on both the TF itself and the context of the comparison. However, the studies also suggest that some TFBS appear to be active consistently across different cellular contexts, and it is not clear what separates such apparently context-independent TFBS from context-dependent sites and whether the genomic context for such sites differs for different TFs.

To address this question, we used ChIP-seq data from two ENCODE cell lines [34] to examine cell-type specific binding sites for seven TFs with known DNA sequence preferences and six transcriptional cofactors with no known sequence preferences. Five of the six cofactors were Polymerase (Pol) III TFs [35], whereas the remaining factors were Pol II TFs. We first show that although both the number of sites and the site overlap differ substantially between TFs, stronger sites, as estimated by ChIP-seq peak height, are generally less cell-type specific than are weak sites. Second, we find that strong sites generally occur more frequently in regulatory regions such as promoters and TFBS clusters and in conserved sequences, compared to weak sites. Moreover, by analyzing cell-type specific chromatin data, we find that strong sites occur more frequently in open chromatin and at histone modifications associated with active promoters, compared to weak sites. Strong sites are also generally more conserved than are weak sites. Third, we show that differences in chromatin can be a reason for cell-type specific TFBS—both at strong and weak sites. We also show that some of the apparent cell-type specific TFBS can be due to differences in genotype that affect sequence motif regions. Finally, by training a machine learning classifier to distinguish common, context-independent sites from cell-type specific sites, we show that site strength and clustering are the most important parameters for identifying context-independent TFBS. Importantly, sites for sequence-specific TFs and sequence-independent cofactors and sites for Pol III and Pol II TFs shared these same characteristics. Thus, our results suggest that context-independent sites are strong, clustered sites in conserved genomic regions.

## Results

### The number of peaks varies between cell-types

**Figure 1:**
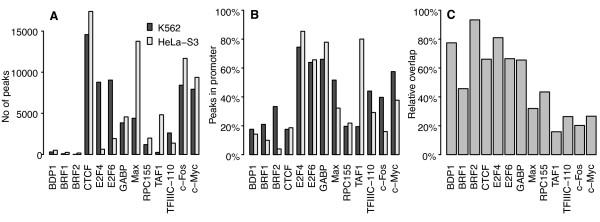
**Discrepancy in peak count and variable peak overlap between cell types. A**) Number of ChIP-seq peak regions per TF in cell types K562 and HeLa-S3. The number of peaks varied for each TF, but there were also big differences between cell types for the same TF. **B**) Percentage of peaks found in promoter regions per TF in K562 and HeLa-S3. A promoter region was defined as the region 2000bp upstream and 200bp downstream of the RefSeq genes’ transcription start sites plus their first intron. On average, a third of all peaks were found in promoter regions. **C**) Peak overlap between K562 and HeLa-S3 relative to potential overlap; that is, the number of peak regions in K562 that overlap by at least one base pair with a peak region in HeLa-S3, divided by the lesser of the peak counts in K562 and HeLa-S3. 33% of K562 and 30% of HeLa-S3 peaks had at least one overlapping peak of the same TF in the other cell type.

Recent genome-wide analyses of TF binding have shown that for a few individual TFs, binding sites can vary substantially between cell-types [29-31]. But how cell-type specific is TF regulation as measured by ChIP-seq data of different commonly expressed transcription factors? To address this question, we used a robust peak detection method [36] on publicly available ChIP-seq data [34] for 13 TFs from two cancer cell lines, K562 and HeLa-S3. (See Additional file 1: Peaks and SNPs for predicted peak regions). Seven of the factors (CTCF, E2F4, E2F6, GABP, Max, c-Fos, and c-Myc) are Pol II factors with specific DNA binding preferences, one (TAF1) is a basal Pol II factor, four (BDP1, BRF1, BRF2, and TFIIIC-110) are general Pol III factors, and one (RPC155) is a Pol III subunit that is enriched at Pol III transcription start sites and has strongly correlated peak heights between K562 and HeLa-S3 [35]. The resulting peaks revealed substantial differences in the number of putative binding sites for different TFs and cell types (Figure 1A). The total number of peaks was similar in both cell types; in sum HeLa-S3 had 11% more predicted peaks than K562, but the peaks were divided unevenly between different TFs in the two cell types. Variable peak counts for different TFs could be expected as the TFs under study serve diverse regulatory roles, but the variability between cell types for the same TF was high.

Nearly half of the K562 peaks (47%) and more than a third of the HeLa-S3 peaks (34%) were associated with promoter regions; specifically, the 2000bp upstream and 200bp downstream of RefSeq genes’ transcription start sites plus their first introns (Figure 1B). We included the first intron in our promoter definition, as binding sites for several TFs are enriched within first introns [37] and such binding sites have been shown to have important regulatory roles for specific genes (for example, the cystic fibrosis transmembrane conductance regulator gene (CFTR) [38]). Despite the variability in the overall number of peaks per TF and cell type, the relative number of peaks mapping to promoter regions for the same TFs were similar between the two cell types (p-value *p *= 0.34 on a two-sided Wilcoxon signed rank test).

### Only a third of peaks are found in both cell types

Given the high variability in putative TFBS, we wondered to what extent these binding sites were cell-type specific. By comparing the genomic loci of peaks across cell types, we found that 33% of K562 and 30% of HeLa-S3 peaks overlapped with at least one peak of the same TF in the other cell type. Taking into account that the relative overlap is limited by the cell type having the fewest peaks for the TF, we found that the potential overlap varied between 16% (TAF1) and 93% (BRF2) (Figure 1C; median 46%). Although the potential overlap was slightly higher for TFs with large differences in peak counts between the cell lines, this apparent trend was not significant (Spearman coefficient 0.20, two-sided p-value *p *= 0.52).

An extreme example of seemingly different binding between cell types is the cell-cycle associated factor E2F4, which had 8780 peaks in K562 but only 631 (7%) in HeLa-S3. A large fraction (81%) of the relatively few E2F4 peaks in HeLa-S3 overlapped with K562 peaks. Others using ChIP-chip have previously found E2F4 to have between 500 and 700 target genes with little cell-type specific binding [1,30]. This could suggest that a majority of the E2F4 peaks in K562 are not functional binding sites, but we cannot exclude that our data are missing several true binding sites in HeLa-S3.

### Peaks in regions associated with tissue-independent regulation have higher overlap than have other regions

**Figure 2:**
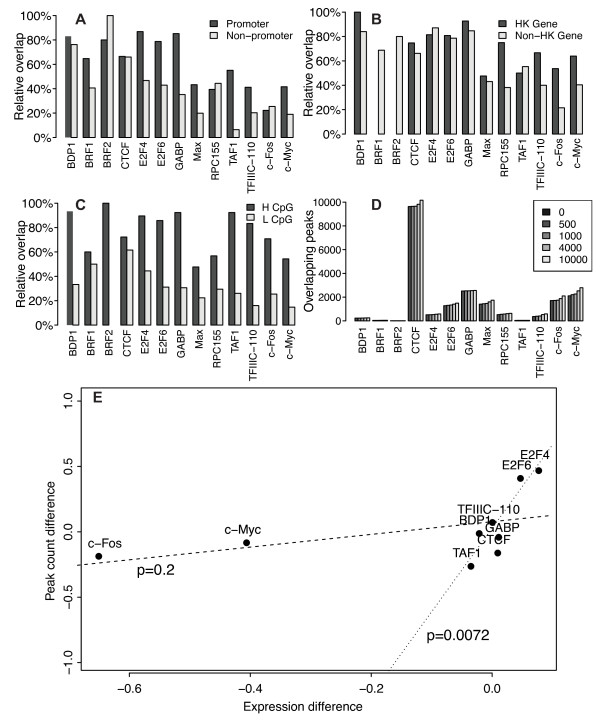
**Explaining regions of increased peak overlap and TF expression difference. A)** Relative overlap (see Figure 1C) of peaks mapping to promoter regions compared with other peaks. Peaks in promoter regions overlap more than peaks in other genomic regions. **B)** Relative overlap of peaks in promoters of housekeeping genes (list from [39]) compared with peaks in other promoters. Peaks in promoters of housekeeping genes overlap more than peaks in promoters of other genes. **C)** Relative overlap of peaks in CpG-rich promoter regions compared with peaks in CpG-poor promoter regions. **D)** Alternative local binding sites. The y-axis shows the number of K562 peaks that overlap with a peak in HeLa-S3 when, one by one, each given peak region in K562 is extended by 0, 500, 1,000, 4,000 and 10,000 bp (half to each side of the peak). The number of overlaps does not increase markedly when considering larger regions surrounding the peaks. **E)** TF expression difference between cell types versus TF peak count difference between cell types. The x-axis gives the difference in number of ENCODE Caltech paired-end RNA-seq reads mapping to a TF gene in K562 versus HeLa-S3 (see Methods). The y-axis gives the difference in number of peaks regions in K562 vs HeLa-S3. Both differences were normalized to the range {-1, 1}. P-values for t-tests on slope of linear regression lines are shown with all TFs included (dashed line; *p *= 0.2) and without the expression outliers c-Fos and c-Myc (dotted line; *p *= 7.2∗10^−3^).

Because of the unexpected differences between the two cell lines for the E2F4 peaks, we wondered whether overlapping peaks were enriched in genomic regions known to have limited tissue-specific activity. A previous study has indicated that TFs primarily mediate cell-type specific regulation through enhancers located far from core promoter regions [9]. In accordance with this, we found that peaks mapping to promoter regions had a significantly higher degree of overlap across cell types than had peaks that did not map to promoter regions (Figure 2A; *p *= 0.0052 for K562 and *p *= 0.0034 for HeLa-S3, one-sided Wilcoxon signed-rank test). Promoter regions were again defined as the region 2000 bp upstream to 200 bp downstream of transcription start sites of RefSeq genes, together with the complete first intron of the genes. Consistent with Pol III factors preferentially associating with regions near functional Pol II promoters [35] three of the four general Pol III TFs showed higher overlap in these mostly Pol II promoters than in the non-promoter regions. Similarly, the subset of Pol II TFs also showed significantly higher overlap in the promoter regions than in other genomic regions (*p *= 0.012 for K562 and *p *= 0.020 for HeLa-S3).

Housekeeping genes tend to maintain similar expression levels across cell types and are therefore also likely to have similar regulation within different cell types. Indeed, the overlap was greater for peaks mapping to promoters of housekeeping genes [39] compared with promoters of other genes (Figure 2B; *p *= 0.0093 for K562 and *p *= 0.0049 for HeLa-S3). Housekeeping genes frequently have high CpG promoters, whereas tissue-specific genes tend to have low CpG promoters [40,41]. Accordingly, peaks in high CpG promoters had significantly higher overlap than had peaks in low CpG promoters (Figure 2C; *p *= 2.4∗10^−4^ for K562 and *p *= 4.9∗10^−4^ for HeLa-S3). Thus, as expected, peaks mapping to tissue-independent regulatory regions showed a higher degree of overlap between cell types than did peaks mapping to other genomic regions.

### Cell-type-specific peaks map to different genomic regions in the cell types

Some of the “missing” overlap could perhaps be explained by alternative transcription factor binding sites, where a binding site active in only one cell type could have another nearby site active in the other cell type. To test this possibility, we counted the number of overlaps when allowing increasingly larger regions surrounding the original peak regions (Figure 2D). We found no striking increase in the number of overlaps, however—even when looking at regions larger than 10,000bp. Consequently, most of the cell-type unique peaks are found at completely different loci in the two cell types.

### Differences in TF expression can partly explain differences in peak counts

The variability in peak numbers could suggest that some TFs have more binding activity in one cell type compared to the other. As one important determinant of binding activity could be TF availability, we looked at how the TF mRNA expression levels, as measured by RNA sequencing, correlated with the number of peaks. We found some, but insignificant, correlation between TF expression and peak numbers (Pearson coefficient *r *= 0.46, two-sided t-test p-value *p *= 0.21 in K562; *r *= 0.54, *p *= 0.11 in HeLa-S3). Using the differences in expression and peak count between the two cell types gave similar results (Figure 2E; *r *= 0.47, *p *= 0.20), but removing the expression outliers c-Fos and c-Myc gave a large and significant correlation (*r *= 0.89, *p *= 7.2∗10^−3^). This suggested that the difference in peak count could partly be related to difference in mRNA expression levels of the TFs.

Of course, the mRNA level does not necessarily alone determine the TF’s actual protein-level [42] and binding activity could be influenced by other variables, such as for example post-translational modifications, chromatin differences, and co-factor availability.

### High peaks are less cell-type specific and more associated with active gene regulation than are low peaks

**Figure 3:**
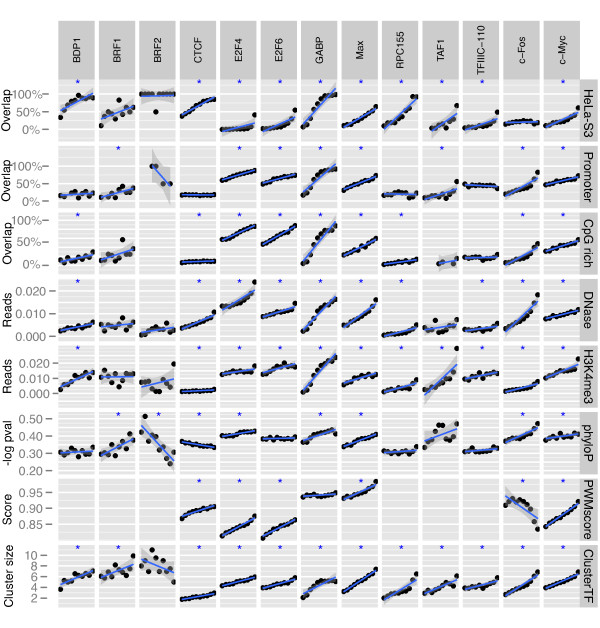
**Higher peaks overlap more and have more consistent support in other data marking regulatory regions.** Peaks in K562 for each TF (panel columns) were binned in 10 equally-sized groups with increasing peak height (ordered along x-axis) and the average values for different genomic characteristics (panel rows) were computed for each group (y-axis). From top to bottom row, the genomic characteristics are: “HeLa-S3”: percentage of peaks in K562 that overlap with a peak in HeLa-S3.; “Promoter”: percentage of peaks that overlap with promoter regions.; “CpG rich”: percentage of peaks that overlap with CpG-rich regions.; “DNase”: average count of DNase-seq reads in peak region—a measure of chromatin accessibility.; “H3K4me3”: average count of H3K4me3 ChIP-seq reads in a peak region—a measure of chromatin activity.; “phyloP”: average phyloP scores in peak region for a 28-way placental mammals multiple alignment—a measure of sequence conservation.; “PWMscore”: average maximal PWM score in peak region (where available).; “ClusterTF”: average number of peaks in peak cluster. See Methods section for definition of promoters, CpG-rich regions, and clusters and for details on other genomic data. The blue line in each panel is a linear regression line between peak height bin and genomic characteristic; the dark gray areas surrounding these lines are 95% confidence intervals; blue stars mark significant regression line slopes (p ≤0.05).

The height of a ChIP-seq peak indicates the TF’s binding activity or strength at the site. Moreover, peak height can give a measure of the likelihood for the TFBS to be active in a given cellular context, as spurious binding events occurring within a fraction of the cells will give low ChIP-seq peaks. We therefore wondered whether cell-type specific binding or binding within regions consistent with regulatory activity, such as promoters or open chromatin, varied with increasing peak height (Figure 3 and Additional file 2: Figure S1 and Additional file 3: Figure S2).

For 10 out of 13 TFs in K562, there were significantly more overlaps among higher peaks compared with lower peaks (Figure 3). The trend was particularly clear for the very highest peaks, save for c-Fos and BRF2—the latter having only 15 peaks in K562. This could mean that cell-type specific binding is generally weaker than the binding in common binding sites, but the observation likely also reflects that spurious binding (stochastic noise) is more prevalent among low peaks.

High peaks were generally more associated with regulatory regions such as promoters (8 TFs significant) and CpG-rich regions (10 TFs significant). The TFs that showed little or no association were not expected to be associated with Pol II promoters; CTCF acts primarily in intergenic and intronic regions [43]), whereas BDP1, BRF1, BRF2, RPC155, and TFIIIC-110 are Pol III factors. The Pol III factors BDP1 and TFIIIC-110 and the Pol III subunit RPC155 did indeed show significant correlation between peak height and overlap with Pol III promoters (Additional file 4: Figure S3; see Methods for description of Pol III promoter regions). Also, nearly all (79 of 81) BRF1 peaks in K562 were associated with Pol III promoter regions, whereas too few BRF2 peaks mapped to the Pol III promoters for a pattern to be evident. High peaks were also associated with increased chromatin accessibility as measured by sensitivity to DNase (10 TFs significant) and a higher enrichment in the histone mark for transcriptionally active promoters H3K4me3 (10 TFs significant), in accordance with previous findings [26].

Even though others have demonstrated poor conservation of binding sites across species for some TFs [30,32], we noted that higher peaks generally resided in regions of higher sequence conservation for many TFs (6 and 4 TFs significant in K562 and HeLa-S3, respectively). The main exception was CTCF, which showed a significant negative correlation between peak height and sequence conservation.

High peaks were also generally more associated with sequence motifs (peaks for 5 of 7 TFs in K562 and 7 of 7 TFs in HeLa-S3 were positively correlated with PWM scores). However, c-Fos peak heights in K562 correlated negatively with PWM scores for the AP-1 motif, which c-Fos recognizes as a dimer with co-factor Jun. One possible explanation could be that competition with Jun homodimers and Jun-ARF2 heterodimers for the AP-1 motif may have pushed c-Fos to bind more in non-canonical motif regions [44]. Alternatively, as higher c-Fos K562 peaks did not have significantly increasing overlap with HeLa-S3 peaks, but did have significantly increasing association with promoters, CpG-rich regions, open chromatin, and sequence conservation, c-Fos or its antibody may have cross-reacted with a different TF in K562.

Using discriminative motif discovery on the 10% highest and 10% lowest c-Fos peaks (see Methods), we found that the highest c-Fos peaks in K562 had motif sequences such as CCAAT and CGCGG, which resemble binding profiles for NF-Y and parts of AP-2, but we did not find any AP-1 binding motif or variant thereof. When using sequences from another TF (CTCF) as negative data and running motif discovery with high and low c-Fos peaks separately as positive data, we again could not find the AP-1 motif in the high peaks (Additional file 5: Figure S4C), whereas the top motif discovered on the lowest peaks was the canonical AP-1 consensus motif (Additional file 5: Figure S4B). We therefore strongly suspect that the highest c-Fos peaks in K562 are the result of some experimental artifact.

As an additional measure of regulatory activity, we looked at how peaks from all TFs clustered together, under the assumption that clustered peaks indicate a region of high regulatory activity [45]. For all TFs save BRF2, higher peaks were significantly correlated with a higher number of TFs in the cluster. Finally, as ChIP-sequencing has a bias for GC-rich fragments [12], we did a separate analysis on a GC-controlled subset of the data. This GC-balanced subset (Additional file 6: Figure S5) had the same trends as the original full dataset (Figure 3). Consequently, any GC-content bias in the ChIP-seq data could not explain the correlations between peak height and different regulatory data seen in Figure 3. Together, these results suggest that high peaks associated with strong and conserved binding sites tend to be cell-type independent or common for many cellular contexts, whereas low peaks associated with weak binding tend to show larger variation between cell types.

### Differences in chromatin state suggest cell-type specific regulation

High TF peaks both had a higher degree of overlap between cell types and were more associated with open chromatin and active transcription, compared to low peaks. However, it was still unclear to what extent cell-type specific differences in high and low peaks were related to differences in chromatin structure or to spurious binding events. We therefore compared chromatin data for K562 and HeLa-S3 in the regions that contained the 30% highest overlapping and non-overlapping peaks and repeated the comparisons for the 30% lowest peaks.

**Figure 4:**
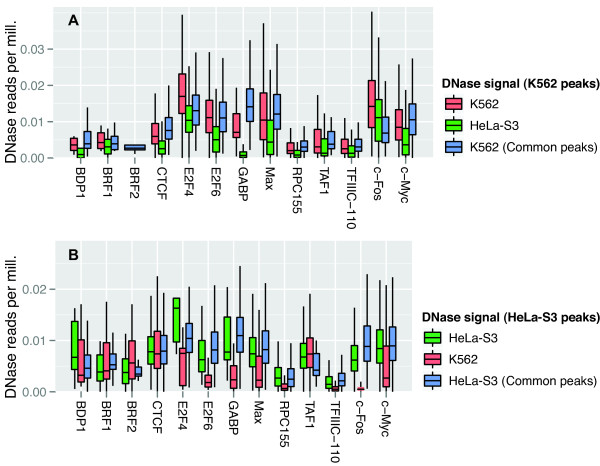
**Differences in chromatin accessibility in cell-type specific peak regions suggest cell-type specific regulation.** Chromatin accessibility as measured by DNase sensitivity for the two cell types in peak regions that are cell-type specific for K562 (A) and HeLa-S3 (B) and in peak regions that are common for both cell types (blue bars in A and B). Only the 30% highest peaks are analyzed. **A)** Box-plots showing for K562-specific TF peak regions, the distributions of DNase-seq signal in K562 (red) and HeLa-S3 (green), and for TF peak regions common to K562 and HeLa-S3, the distribution of DNase-seq signal in K562 (blue). The DNase-seq signal was the read per million-normalized number of reads mapping to each peak region divided by the region length. BRF2 has only one box-plot as all BRF2 peaks overlapped in the two cell lines. **B)** Similar data as in (A), but for HeLa-S3-specific peak regions the DNase-seq signal in HeLa-S3 (green) and K562 (red) and for peak regions common to K562 and HeLa-S3 the DNase-seq signal in HeLa-S3 (blue). Most of the TFs have comparable DNase-seq signals at the common peak regions in the two cell lines (compare blue bars in A and B). Moreover, most of the TFs show symmetric signals at the cell-type specific peak regions, such that the regions that are specific for K562 have higher DNase-seq signals in K562 than in HeLa-S3 (A) and vice versa (B).

**Figure 5:**
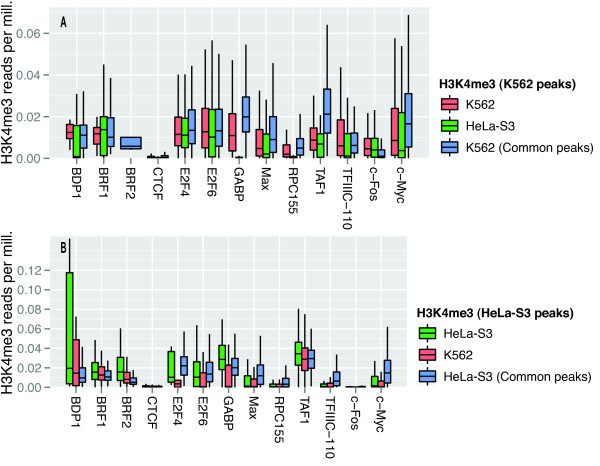
**Differences in H3K4me3 signal in cell-type specific peak regions suggest cell-type specific regulation.** H3K4me3 enrichment for the two cell types in peak regions that are cell-type specific for K562 (A) and HeLa-S3 (B) and in peak regions that are common for both cell types (A and B). Only the 30% highest peaks are analyzed. **A)** Box-plots showing for K562-specific TF peak regions, the distributions of H3K4me3 ChIP-seq signal in K562 (red) and HeLa-S3 (green), and for TF peak regions common to K562 and HeLa-S3, the distribution of H3K4me3 ChIP-seq signal in K562 (blue). The H3K4me3 ChIP-seq signal was the read per million-normalized number of reads mapping to each peak region divided by the region length. BRF2 has only one box-plot as all BRF2 peaks overlapped in the two cell lines. **B)** Similar data as in (A), but for HeLa-S3-specific peak regions the H3K4me3 ChIP-seq signal in HeLa-S3 (green) and K562 (red) and for peak regions common to K562 and HeLa-S3 the H3K4me3 ChIP-seq signal in HeLa-S3 (blue). The H3K4me3 ChIP-seq data show the same patterns as the DNase-seq data (see Figure 4).

In general, chromatin was more accessible and had a higher signal for the active histone mark H3K4me3 in the cell type where the cell-type specific peak regions were found, compared to the same regions in the other cell type (Figures 4 and 5). Differences in chromatin accessibility, as measured by DNase sensitivity, were significant on a Kolmogorov-Smirnov test for 10 of 13 TFs in K562 and for 9 TFs in HeLa-S3. Differences in H3K4me3 signal were significant for 10 TFs in K562 and 9 in HeLa-S3.

For the lowest 30% of peaks we expected to see smaller differences between the chromatin state in peak and non-peak regions, as a smaller difference would be consistent with increased stochastic noise due to more spurious binding within the peak data. Indeed, although the differences in DNase sensitivity and H3K4me3 signal were more consistent for the weak than for the strong peaks (11 TFs significant for DNase both in K562 and HeLa-S3; 11 TFs in K562 and 13 TFs in HeLa-S3 for H3K4me3; Additional file 7: Figure S6 and Additional file 8: Figure S7), the median difference in chromatin accessibility and H3K4me3 signal was greater for higher peaks (Additional file 9: Figure S8). It is therefore likely that a larger fraction of the low than of the high cell-type specific peaks is a result of spurious binding events or stochastic noise in the data.

### Different genotypes can contribute to binding differences

Another factor that could contribute to differences in binding between cell types is single nucleotide polymorphisms (SNPs), as K562 and HeLa-S3 are established from two different individuals with different genotypes. We aligned available DNA sequencing data for K562 and HeLa-S3 to the reference genome (hg18) and by using SNPs from the HapMap database and the aligned sequence reads, we calculated the most likely genotype for each cell type for the SNPs in peak regions (see Methods).

In total, 12% of the peaks harboured at least one SNP. Relative to peak count, more overlapping peaks had SNPs (13.9%) than K562-specific (11.8%) or HeLa-S3-specific (12.1%) peaks. But a relatively higher number of SNPs in cell-type specific peaks were homozygous with different alleles in the cell types (18%, 17.8%, and 10% for K562-specific, HeLa-S3-specific, and overlapping peak SNPs, respectively).

**Figure 6:**
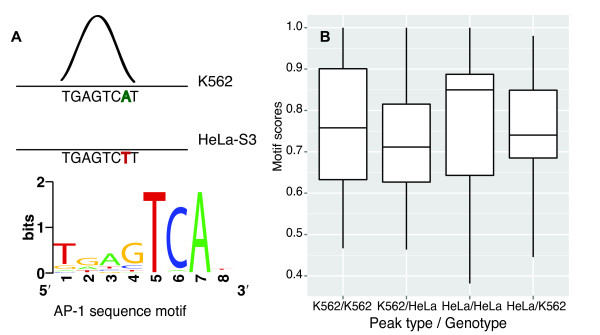
**Genotype differences in sequence motifs can give cell-type specific peaks.A)** A specific example of how different alleles can create differences in sequence motifs, possibly causing cell-type specific TF-binding. SNP rs7138374 (chr12:130,642,970) is located at the highly conserved position 7 in the highest scoring AP-1 sequence motif in a K562-specific c-Fos peak region. K562 is homozygous for the A allele (green letter) and has a peak (illustrated here by a curve; top of panel). HeLa-S3 is homozygous for the T allele (red letter), which disrupts the motif, and has no peak (illustrated by absence of curve; middle of panel). The bottom part of the panel shows the sequence logo for the AP-1 sequence motif. **B)** A comparison of PWM motif score distributions in K562- and HeLa-S3-specific peaks that contain SNPs in the highest-scoring sequence motif regions in the peaks, and where these SNPs are homozygous but have different alleles in the two cell types. The two leftmost box-plots compare for the K562-specific peaks that contain such homozygous SNPs, the PWM motif scores for the K562 and HeLa-S3 genotypes (K562/K562 and K562/HeLa, respectively); the two rightmost box-plots compare for the HeLa-S3-specific peaks that contain such homozygous SNPs, the motif scores for the HeLa-S3 and K562 genotypes (HeLa/HeLa and HeLa/K562, respectively). The K562-specific peaks have significantly higher motif scores for the K562 genotype (K562/K562) than for the HeLa-S3 genotype (K562/HeLa), whereas the HeLa-S3-specific peaks have significantly higher motif scores for the HeLa-S3 genotype (HeLa/HeLa) than for the K562 genotype (HeLa/K562; *p *= 1.9∗10^−4^and *p *= 1.1∗10^−5^, one-sided paired t-tests for K562- and HeLa-specific peaks, respectively).

We reasoned that the SNPs that were homozygous for one allele in one cell type and homozygous for another allele in the other cell type could affect TF binding if the SNP resided in a high-scoring TF motif (Figure 6A). Indeed, the peaks that were cell-type specific and contained such SNPs had significantly higher motif scores in the cell type having the peak compared with the other cell type (Figure 6B; *p *= 1.9∗10^−4^ and *p *= 1.1∗10^−5^ on one-sided paired t-tests for K562 and HeLa-specific peaks, respectively). Among 73 K562-specific peaks that fitted the SNP and motif criteria stated above, 49 had higher motif scores in K562 and 24 had higher scores in HeLa-S3. Among 82 HeLa-S3-specific peaks, 60 had higher motif scores in HeLa-S3 and 22 in K562.

Genotype-related differences were also evident in peaks common between the two cell types. Specifically, for the SNPs that had different homozygous genotypes and were located in high-scoring motif regions, the difference in motif score between the genotypes correlated positively with the difference in peak height between the cell lines (*r *= 0.22, *p *= 0.09). This correlation was especially strong and significant for the 10% highest peaks, both for the peaks that had SNPs in high scoring motif regions (*r *= 0.59, *p *= 9.9∗10^−3^) and for the peaks that had SNPs in any location within the peak region (*r *= 0.43, *p *= 4.5∗10^−3^). Thus, differences in genotypes do affect TF binding and can explain some of the binding site differences between cell lines.

### Peak height and clustering are the most important factors for cell-type specificity

Our results so far indicated that the cell-type specificity we observed among ChIP-seq peaks was related to a number of different factors that included peak height and locus, sequence properties of the peak region, and the region’s cell-type specific chromatin context. But which factors are the most important indicators of cell-type specific TF binding and to what extent do combinations of factors determine cell-type specific binding?

To address these questions, we used a machine learning approach. Specifically, we created support vector machine (SVM) classifiers [46] to separate cell-type specific peaks from peaks that are found in multiple cell types. The SVM’s feature set for each peak included both cell-type specific information such as chromatin state, and cell-type independent information such as sequence conservation; see Table 1 for a full list of features. Using peaks from K562 as a reference and comparing overlap and cell-type specific data with the corresponding regions in HeLa-S3, we trained for each TF one SVM classifier to predict the K562 peaks that overlapped with HeLa-S3 peaks. We then used 10-fold stratified cross-validation to estimate the SVM classifiers’ predictive performance in terms of their receiver operating characteristic (ROC) curve and ROC-score (Additional file 10: Figure S9A).

**Table 1:** SVM Features

**No**	**Name**	**Group**	**Comment**
1	Height	Height	Peak height (percentiles)
2	Length	Length	Peak length
3	Promoter	Promoter	Overlap with promoter (boolean)
4	TSS dist	TSS dist	Dist. to closest transcription start site (max 20.000)
5	Cluster TFs	Cluster	Number of TFs in overlapping cluster
6	Cluster avg height	Cluster	Avg peak height in overlapping cluster
7	Chromatin avg	Chromatin	Avg DNase signal in two cell types
8	Chromatin diff	Chromatin	DNase signal diff between two cell types
9	H3K4me3 avg	H3K4me3	Avg H3K4me3 signal in two cell types
10	H3K4me3 diff	H3K4me3	H3K4me3 signal diff between two cell types
11	H3K27me3 avg	H3K27me3	Avg H3K27me3 signal in two cell types
12	H3K27me3 diff	H3K27me3	H3K27me3 signal diff between two cell types
13	CpG freq	CpG	CpG frequency in peak region sequence
14	High CpG	CpG	Equal to 1 if sequence is high in CpG
15	Low CpG	CpG	Equal to 1 if sequence is low in CpG
16	PhyloP	PhyloP	PhyloP conservation score in sequence region

The SVM predictors were given these data as input for training and were classifying peaks as overlapping or not (that is, cell-type specific).

**Figure 7:**
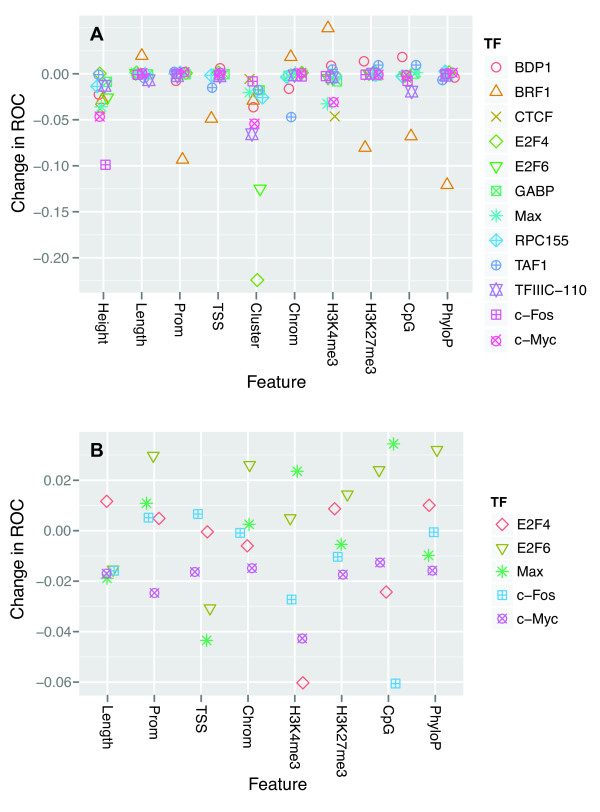
**Most important features for classification of peak cell-type specificity.A)** Difference in average 10-fold crossvalidated ROC-score for each TF SVM classifier after removing all features within a feature group (see Table 1), compared to including all features. Y-axis shows the change in ROC-score after removing the corresponding feature for the given TF. Removing peak clustering or peak height gives a decrease in ROC-score for most TFs. **B)** As (A), but after removing confounding factors from the analysis. Specifically, only the 10% highest and 20% most clustered peaks were used, and peak height and cluster features were removed from the SVM training and test datasets. Only the five TFs that had more than 100 remaining peaks in both the overlapping and the cell-type specific datasets were considered. The importance of features varies for each TF. Distance to TSS seems to be more informative than the binary promoter feature, and removal of the cell-type specific mark for active chromatin (H3K4me3) gives the highest performance penalty overall.

To assess which SVM features were most important for correct classification, we removed a single feature group at a time from the SVM and measured how this affected the SVM’s cross-validation performance (Figure 7A). Although the results were somewhat different for each combination of TF and feature, one result was clear: peak clustering and peak height were the only features that had a strong positive impact on the prediction for the majority of the TFs. Peak clustering was particularly important for classifying E2F4 and E2F6 peaks, whereas information on peak height was important to most TFs. As for individual TFs, the two TFs with the fewest peaks in their datasets, BDP1 and BRF1, had larger score variation than the other TFs. As a result, the TFs appeared to benefit from removing some features. Finally, removing information about the distance to the closest transcription start site (TSS) had an expected negative impact on TAF1, which is a core component of the Pol II basal transcription factor TFIID [47].

Given the major importance of peak height and clustering for classifier performance, and in light of previous results (Figure 3), we reasoned that including these features made it difficult to assess any importance of other features. We also suspected that some of the features could contain redundant information, as removing single features had relatively little impact on performance. We therefore removed peak height and clustering from the dataset and grouped features into the following three groups: cell-type specific features (chromatin accessibility and histone modification status), promoter and sequence-associated features (promoter, TSS distance, CpG content), and conservation feature (phyloP). The results (Additional file 11: Figure S10) showed that cell-type specific and promoter-associated features were important for, respectively, most (all except E2F6) and some (CTCF, E2F6, c-Fos, and c-Myc) of the sequence-dependent TFs, whereas only promoter-associated features were important for some of the general TFs (TFIIIC-110 and RPC155). Sequence conservation, however, was not an important factor for identifying cell-type specific peaks in this dataset.

Peak height and clustering were the dominant factors in explaining cell-type specificity and could therefore confound the effects of other features when analyzing the total set of all peaks. We therefore did a separate analysis of the highest and most clustered peaks, as differences between cell types for these peaks should be due to other factors than peak height and clustering. Specifically, we first identified the subset of peaks that were among the 10% highest and that also occurred among the top 20% largest peak clusters; that is, in clusters containing peaks from six or more TFs. Second, we did a feature removal analysis on the five TFs that had more than 100 remaining peaks in both the overlapping and the cell-type specific datasets. Although none of the features on this reduced dataset showed the same consistent pattern as the height and cluster features did on the complete dataset, five of the eight features (Length, TSS, H3K4me3, H3K27me3, and CpG) had a positive impact on the prediction for the majority of the five TFs (Figure 7B). Moreover, only one feature (Prom) had a negative impact on the prediction for the majority of the TFs. These results confirmed that additional factors beside peak height and clustering, including cell-type specific data on histone modifications, are important for identifying cell-type specific TF binding.

### Models for identifying cell-type specific peaks are consistent between cell lines

**Figure 8:**
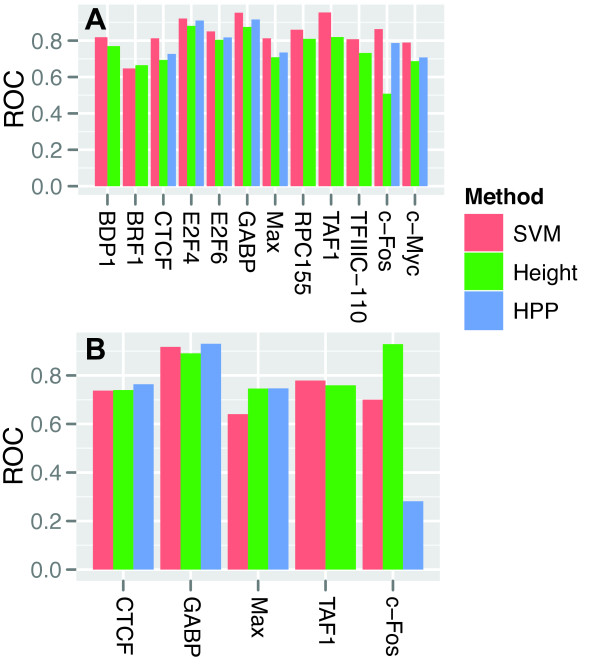
**Best prediction performance with all features.A)** ROC-score for each TF classifier using the SVM model with all features (SVM), with peak height only (Height), and with peak height, phyloP conservation and PWM score (HPP), after 10-fold stratified cross-validation on the dataset consisting of K562 and HeLa-S3 peaks. **B)** ROC-score after 10-fold stratified cross-validation when training classifiers on K562 and HeLa-S3 peaks, and then testing for overlap with peaks from a third cell type, GM12878.

The feature analysis suggested that even though peak height alone was a critical factor for identifying cell-type specific peaks, other features could also help predictions. To further assess the effect of adding additional features, we therefore developed two reference models, which we refer to as Height and HPP. These models were also based on SVMs, but contained fewer features; Height used only peak height as its single feature and HPP used peak height, phyloP conservation score, and PWM score (Figure 8A; see Additional file 10: Figure S9A for the ROC curves).

Consistent with the feature analyses, the SVM model that included all features (termed SVM in Figure 8), was significantly better than the two reference models (*p *= 4.9∗10^−4^and *p *= 7.8∗10^−3^ for Height and HPP, respectively, on a Wilcoxon signed-rank test). The HPP model was also significantly better than Height (*p *= 7.8∗10^−3^). The Height model only had a higher ROC-score than the full SVM model on the BRF1 dataset. This is likely due to a low number of training examples for the more complicated SVM method, as there were only 81 BRF1 peaks in K562. Consistent with previous results (see c-Fos peak overlap in Figure 3), peak height alone was insufficient to predict overlapping c-Fos peaks.

So far we had tested how the models performed when classifying new unseen peaks from the same cell types (K562 and HeLa-S3) used during training, but would the models generalize to a different cell type? To address this question, we used a stratified 10-fold cross-validation procedure where we first trained the models on a subset of K562 peaks that were common and cell-type specific compared with HeLa-S3 peaks. We then tested the models on a different subset of K562 peaks that were common and cell-type specific compared to peaks in the GM12878 ENCODE cell line; that is, we trained the models on K562 and HeLa-S3 data and tested the models on K562 and GM12878 data. Importantly, none of the peaks in these train and test sets overlapped and the cell-type specific features (Chromatin, H3K4me3, and H3K27me3) were derived from two different cell types in the train (HeLa-S3) and test (GM12878) sets. We trained and tested the models on the five TFs with ChIP-seq data available in all three cell types.

Whereas the Height and HPP models had similar performance in the new cell type (*p *= 0.22 and *p *= 0.43, respectively; one-sided Wilcoxon signed rank test), the full SVM model had a significant drop in performance (*p *= 0.031); see Figure 8B and Additional file 10: Figure S9B. This drop resulted in the full SVM model having lower ROC-scores than the Height model had on three of the five TFs. The largest differences between the SVM and Height models were on the Max and c-Fos data, suggesting that the SVM model was over-fitted on these particular TFs. Indeed for c-Fos, the different features in the SVM were more inconsistent between the three cell types than for the other TFs (7 of 14 feature trends differed, compared with 5 of 12 for TAF1 and at most 2 of 14 differing trends for the three other TFs; compare Figure 3, Additional file 2: Figure S1, and Additional file 3: Figure S2).

These inconsistencies between the cell types also strongly affected the Height and HPP models on the c-Fos data. As already mentioned, Height could not predict c-Fos peaks that overlapped between K562 and HeLa-S3, whereas the HPP model trained on K562 and HeLa-S3 data could not predict peaks that overlapped in K562 and GM12878. Of the five TFs, c-Fos had the least consistent binding sites between the three cell types (Additional file 12: Figure S11). c-Fos is less specific in recognizing the AP-1 site compared to dimer-partners Jun and ATF2 [44], so some of the differences could be because c-Fos used different co-factors in the three cells. This explanation is consistent with c-Fos having three different trends in peak heights and PWM scores for the three cell lines. Moreover, Jun expression level was four times as high in HeLa-S3 as in K562, and the strong c-Fos peaks in HeLa-S3 had good AP-1 consensus motifs compared to weaker peaks (Additional file 2: Figure S1). However, as previously mentioned, we cannot exclude that c-Fos or its antibody had cross-reacted with a different TF in K562. Apart from c-Fos, however, the models could consistently separate cell-type specific from cell-type independent TFBS.

## Discussion

There is limited knowledge on how regulation by transcription factors in general varies between different cellular conditions, and what causes specific transcription factor binding. Most newer approaches for inferring gene regulatory networks rely on experimental data that partly limit the inferred networks to a specific cellular context. While this makes it possible to focus on the interactions that are active in the particular context under study, it also means that it can be difficult to completely map the general regulatory network by using these approaches [21]. Because ChIP-seq is increasingly used for inferring gene regulatory networks, and is also used to validate predictions of other methods [48], it is important to understand to what extent ChIP-seq data are limited to a specific cellular context. Here we have investigated ChIP-seq data for a number of TFs and examined possible causes of observed differences in binding.

Our initial analysis indicated that in general, the number and location of binding sites as given by ChIP-seq peak data varies substantially between HeLa-S3 and K562—even for the same TF. The average peak overlap of 30% we found is in accordance with previous findings on TF binding specificity among different cell-lines [31], and we note that similar levels of overlap have been found for regions of open chromatin between a number of different cell lines [28].

These binding differences can partly be explained by cell-type specific regulation. Specifically, our results show that chromatin accessibility, histone modifications, and genetic variations in peak regions can explain some of the cell-type specific peaks. Another factor that could establish alternative binding contexts is a difference in availability of TF co-factors across cell types [11]. Although we found significant differences in co-factor expression and PWM score distributions between the cell types (see Additional file 13: Table S1 and Methods), because of limited and noisy PWM and interaction data it is difficult to quantify the overall effect of co-factor differences. Stochastic noise does appear, however, also to be a major factor in causing binding differences. Specifically, we found that peak height and additional TF binding events (clustering) are the most important features for discriminating between overlapping and cell-type specific peaks. When focusing on the 10% highest and 20% most clustered peaks, the average overlap increased to 58% and 68% for the K562 and HeLa-S3 peaks and open chromatin and histone modifications were important for separating cell-type specific from common peaks. Low peaks, in contrast, were less associated with features supporting regulatory activity (Figure 3), including condition-independent features such as PWM score and sequence conservation, indicating that a larger fraction of the low peaks than of the high peaks represents stochastic noise or spurious binding events.

Both high and low affinity binding sites are, however, known to have biological importance in regulating gene expression [49,50]. Merely setting a high cut-off on peak height to reduce noise [51] will therefore risk losing many functional sites. Conversely, using peak height to infer whether a candidate TFBS will have binding activity in other cellular contexts risks introducing false positive regulatory interactions. Our SVM classifier can partly solve both these problems, as the SVM is a better alternative than peak height alone for identifying TFBS that are common between cell types, when sufficient training data are available. Alternatively, combining peak height with evolutionary conservation and TF motif scores (our HPP model) gives good predictions when less data are available. Being less complex than the SVM model, the HPP model also is less prone to overfitting than the SVM model, as seen when the models were tested on a third cell type. As all three methods for finding common TFBS sites are data-driven, however, all three methods can suffer from inconsistent data, such as the c-Fos datasets. Apart from c-Fos and TFs with limited data, both the SVM and HPP models outperformed the simple peak height-based model for finding consistent TFBS. These models can therefore also be used to remove noise and identify high-confidence TFBS in ChIP-seq data.

Peak height and clustering are the most important features for the SVM. Consequently, if context-specific peaks are indeed much more noisy than context-indifferent peaks, the SVM classifiers are perhaps to a greater degree learning to differentiate between real binding sites and noise, and to a lesser degree learning to differentiate between context-independent and context-dependent binding sites.

Our results show that low peaks are less consistent than high peaks, but what are then potential causes for these inconstancies? Weak, apparently random TF binding, non-specific antibody binding, and sequencing errors combined with peak calling artefacts [12,36] can contribute random noise, but one intriguing possible contribution is cell culture subpopulations that have slightly different cellular contexts. Cell cultures, such as the three cultures used in the ChIP-seq experiments we based our analyses on, are generally a heterogenous mix of individual cells in slightly different biological states. For example, unsynchronized HeLa cells growing in culture consist of different subpopulations of cells that are in different cell cycle phases and have slightly different transcriptional states [52]. Population-wide measurements, such as ChIP-seq data, from such unsynchronized cell populations represent an average of these individual states. Consequently, binding events that are biologically consistent but only occur within a small fraction of the cells, because these cells are in a transient transcriptional state, will give low or undetectable ChIP-seq peaks that can appear to be random events. Supporting this explanation, the 30% lowest cell-type specific peaks in the ChIP-seq data showed more consistent differences in DNase sensitivity and H3K4me3 signal between the cell lines than the 30% highest peaks showed (Figures 4 and 5 and Additional file 7: Figure S6 and Additional file 8:Figure S7), even though the differences in DNase sensitivity and H3K4me3 signal were smaller for the low than for the high peaks (Additional file 9: Figure S8). Small but consistent differences in DNase sensitivity and H3K4me3 signal together with low TF peaks indicate that these TF binding events occur within small but consistent subpopulations of the cell cultures. More focused experiments in synchronized cell cultures may therefore be necessary to completely and correctly map context-dependent regulatory networks.

## Conclusions

Many methods for inferring gene regulatory networks are based on ChIP-seq-predicted transcription factor binding sites, but little is known about how specific these data are to the experimental context. Here we find that on average a third of ChIP-seq predicted transcription factor binding sites overlap between the cell lines K562 and HeLa-S3. This number is in accordance with previous findings on cell-type specificity and suggests that regulatory networks inferred uncritically from ChIP-seq data will, in general, generalize poorly to different cellular contexts. However, some subgroups of peaks are less context-specific than others. Specifically, peaks in regions associated with tissue-independent regulation, such as CpG-rich promoter regions, and more generally, high and clustered peaks associated with strong and conserved binding sites, tend to be more cell-type independent.

Even though some of the discrepancy between the two cell types can be caused by cell-type specific regulation of chromatin and co-factors, the results indicate that most of the discrepancy—especially among the low peaks—is due to stochastic noise in the ChIP-seq data. Our results do, however, not exclude that different sub-populations contribute to the discrepancy. Specifically, the small but consistent differences in DNase sensitivity and H3K3me3 signal we observed for the 30% lowest peaks are consistent with distinct sub-populations within the data. Nevertheless, context-independent attributes, such as PWM score and sequence conservation, are more associated with stronger and more overlapping peaks than with weak and isolated peaks.

Moreover, both sequence-specific TFs and sequence-independent cofactors shared the same characteristic of stronger peaks being more cell-type independent. Indeed, even the sites for the Pol III subunit RPC155, which is enriched at Pol III promoters and shows highly correlated peak heights between different cell lines [35], were more cell-type independent and more associated with Pol III promoters when the sites corresponded to strong compared to weak peaks. Consequently, it is likely that stochastic binding events instead of consistent cell culture sub-populations explain most of the observed cell-type specific binding events for the low and isolated peaks. Accordingly, methods that try to build context-independent gene regulatory networks from ChIP data could benefit from focusing on high and clustered peaks in conserved genomic regions. Our SVM method can help identify such peaks.

It might be a while before binding sites of most TFs are mapped in a majority of tissues and cell lines. Until then, any available ChIP-seq data, however noisy and condition-specific, is likely to serve as our best source of information on the location and context of regulatory elements.

## Methods

### ChIP-seq data and peak calling

The ChIP-seq data were based on public genome-wide ChIP-seq datasets from the ENCODE project [34], available from the UCSC Genome Browser as Yale TFBS and HAIB TFBS tracks [53]. Data was downloaded for 13 TFs (see Figure 1) for cell types K562 and HeLa-S3, and for 5 TFs (CTCF, GABP, Max, TAF1, c-Fos) for GM12878. The raw tagcount data was then processed by our own peak detection method [36] which we briefly describe here:

ChIP-seq peaks were identified in sample and replicate data by two different peak-finder programs, MACS [54] and SISSRs [55]. Both programs were run using independent background samples to correct for biases in the background tag distribution. To reduce the number of false and spurious peaks identified, only peaks identified by both programs, and in the replicate for MACS, were used in the benchmark. Peak regions were then shortened to 100-400bp by a peak-trimming procedure to reflect the resolution in ChIP-seq data.

### Defining overlap regions and peak clusters

Data from the cell type K562 was used as a reference in further analysis unless stated otherwise. A peak in a cell type was defined as overlapping with another peak in a different cell type if the two peak regions shared at least one base pair. Relative overlap was calculated as the number of overlaps divided by the number of peaks in the cell type (K562 or HeLa-S3) having fewest peaks for the given TF. Promoter regions were defined as the region 2000 bp upstream to 200 bp downstream of transcription start sites of RefSeq genes (as of October 22. 2009), together with the first intron of the gene. Pol III promoters were defined as the regions -2000bp upstream and +200bp downstream of tRNA transcription start sites from the tRNAscan-SE Genomic tRNA Database [56] (downloaded June 11. 2012).

CpG frequencies and CpG region types were computed as in [41]. Specifically, CpG-rich regions were defined as regions having at least one subregion of length 500 bp with GC content > 55*%* and CpG frequency > 75*%*; CpG poor regions having CpG frequency < 48*%*. The list of housekeeping genes were downloaded from [39].

Peaks were clustered by first extending each peak region to a total length of 2000bp. Peaks overlapping within the extended region were then identified as belonging to the same cluster [45].

### TF expression

Paired-end RNA-seq reads were downloaded from the ENCODE Caltech RNA-Seq track in the UCSC Genome Browser. Both available replicates were used and the number of reads mapping to each RefSeq exon was counted. The count was normalized on exon length and averaged, to get the expression for a given RefSeq. Counts were also normalized on total number of reads within one experiment when comparing across experiments. The expression for a TF was averaged from all RefSeqs whose gene symbol mapped to the TF and had at least 1 read mapping to an exon.

### Peak binning, chromatin differences, and PWM score

To investigate how peak height correlated with other genomic features, we binned peaks in 10 approximately equally-sized groups according to peak height (i.e. number of tags mapping to peak region). Each peak got a measure of chromatin accessibility using data on DNaseI hypersensitivity from the ENCODE Open Chromatin track available from the UCSC Genome Browser: Using the tag files with aligned reads from the DNaseI hypersensitivity sequencing (DNase-seq) experiments, we counted the number of tags that overlapped with each peak region and divided by the peak region length and total number of tags (in millions) in the experiment to control for variation in peak region length and differences in sequencing depth between experiments. We used version 2 of the dataset and all available experimental replicates. Likewise, data on H3K4me3 and H3K27me3 was taken from the ENCODE UW Histone ChIP track. The average phyloP score in each peak region was calculated from the phyloP28Way placental mammals multiple alignment scores, also available from UCSC.

An in-house developed program was used to calculate PWM scores [48]. The maximal PWM score on the sequence from both strands in the peak region was used as the PWM score of the peak. The PWMs were taken from release 2008.2 of the Transfac Professional Database [57] and the Jaspar database [58], downloaded on October 12th, 2009. A pseudo-count of 1 was added to each base position to avoid any potential zeros when calculating the log-odds score. The background distribution of each base was calculated by counting the number of times each base occurred in the whole hg18 genome and dividing by the sum for all four bases. See Additional file 14: Table S2 for a list of which PWMs were used.

### Genotyping and SNP analysis

Raw reads from ENCODE [34] ChIP-seq data for K562 and HeLa-S3 were downloaded from the Yale TFBS and HAIB TFBS tracks available in the UCSC Genome Browser [53]. The reads were aligned to the hg18 [59] genome using Novoalign [60]. Novoalign supports alignment using IUPAC ambiguity codes. To account for allele bias, we made a “masked” version of the reference genome with these ambiguity codes at locations of SNPs in dbSNP [61]. As the “reference genome” input to Novoalign, we used the regions consisting of the peak regions flanked by 30 nucleotides upstream and downstream in the masked reference genome. Reads with low alignment quality or multiple alignment positions were filtered out. SAMtools [62] was used to merge files with aligned reads to do genotype calling. Genotypes were called using SAMtools’ default settings. SAMtools takes into account the number of reads, quality of reads, and quality of single bases at the position of variation. SNPs were filtered using annotated SNPs from the HapMap database [63]. Allele frequencies were calculated based on ratio of reads with reference and alternate allele. Additional file 1: Peaks and SNPs includes all peak regions and the SNPs mapping to the peak regions.

### Cofactors

A list of potential co-factors for each TF was made by combining data from the Fantom consortium [11] and the annotated interactions in the Transfac database [57]. Differentially expressed co-factors were defined as those co-factors being among the top 5% most differentially expressed TFs in the list. We applied the Kolmogorov-Smirnov test for difference (*p*≤0.05) in the distribution of PWM scores for the known co-factor PWMs in a 1000 bp region surrounding the cell-type specific peaks of K562 and HeLa-S3.

Discriminative motif discovery in 500 bp regions centered on cell-type specific peaks was done with DREME [64], using an E-value cutoff 0.01 and with 4 as the minimal k-mer length. Motifs were matched against Transfac [57] and Jaspar databases [58] with TomTom [65], using threshold 0.1 and with 3 as minimal overlap length. All gene symbols from the matched PWMs in the Transfac Factor table or Jaspar were filtered to keep only approved gene symbols as defined by HGNC [66]. The resulting gene symbols were then matched against our list of potential co-factors.

### SVM features and classification setup

The list of features for the SVM method is available in Table 1. Peak height was transformed into percentiles (separately for each TF and cell-type grouping). Peak length was the length in nucleotides of the trimmed ChIP-seq peak. The promoter feature was 1 if the peak overlapped at least 1 bp with the promoter region (defined above), or else 0. TSS distance was the maximum of the distance to the closest TSS and 20.000, so as to limit the range of the feature. The clustering features included a count of the number of peaks in the peak cluster (if any) and average peak height in cluster. See above for details on how the clustering was done.

The chromatin and histone data for each peak were calculated as explained above. For the features we calculated the average read count in the peak region in two cell types, and also the difference in read count between the cell types. CpG features were computed as explained above (in “Defining overlap regions and peak clusters”). The phyloP feature was calculated for each peak region by using a weighted average of all the signal values that overlapped with a peak region, weighting each value by the length of the signal region.

Two 10-fold crossvalidation tests were performed on all three methods using peaks from the K562 cell type. The first test investigated the ability to recognize which K562 peaks overlapped with HeLa-S3 peaks when trained and tested on different parts of the K562 dataset. The second tested how the methods could predict which K562 peaks overlapped with GM12878 peaks when being trained on data from comparing K562 peaks with HeLa-S3.

When doing feature elimination on single features, all features within a single feature group were removed from the dataset before training and testing. Feature elimination was also done with larger groups of features, as explained in Results. All of the tested methods were based on the same Support Vector Machine setup for consistency. The classification was performed using a combination of own code and the PyML machine learning framework [46,67]. We chose to use an SVM-based classifier because of our previous experience with the algorithm, and because SVMs have been used extensively and with success on high-dimensional and large classification problems in computational biology [46].

### Availability

The ChIP-seq peak region data are available upon request.

## Competing interests

The authors declare that they have no competing interests.

## Author’s contributions

Analyzed the data and drafted the article (TH). Created the peak region datasets and cluster regions from ChIP-seq tag count data (MR). Calculated genotype for SNPs in the cell types (RM). Conceived the study (TH, PS, FD). Helped prepare the final article and supervised the work (PS). All authors read and approved the final manuscript.

## Supplementary Material

Additional file 1: Peaks and SNPs This tab-separated file includes all peaks and the SNPs mapping to the peak regions. The fields are peakID, cell type, TF, chromosome, peak start, peak stop, height, overlapK562, overlapHeLa-S3, overlapGM12878, overlapPromoter, SNPs. The SNP field is further delimited by a dash (−) for each SNP in the peak region. Each SNP is described by ID, genotype (0=as reference, 1=alternate allelle), genotyping quality score estimated by SAMtools [62] (Q), frequency of alternate allelle (AF), and number of reads containing position (DP). The values describing a SNP are separated by semicolons. Click here for file

Additional file 2: Figure S1 Higher peaks in HeLa-S3 have more consistent support in other data marking regulatory regions. As Figure 3, but for HeLa-S3 peaks instead of K562. Overlap is here measured as percentage of HeLa-S3 peaks that overlap with K562 peaks. Click here for file

Additional file 3: Figure S2 Higher peaks in GM12878 have more consistent support in other data marking regulatory regions. As Figure 3, but for GM12878 peaks. Overlap is here measured as percentage of GM12878 peaks that overlap with K562 peaks. Click here for file

Additional file 4: Figure S3 Pol III promoter overlap correlates with peak height for Pol III-associated factors. Similar to Promoter data in Figure 3, but for overlap with Pol III promoters, (defined as the regions -2000bp upstream and +200bp downstream of tRNA transcription start sites from the tRNAscan-SE Genomic tRNA Database [56]). The Pol III-associated factors and subunits BDP1, RPC155 and TFIIIC-110 all show significant correlation between peak height and promoter overlap. Click here for file

Additional file 5: Figure S4 Different motifs in low and high c-Fos peaks. **A**) The canonical AP-1 motif taken from the Transfac database [57] (matrix identifier V$AP1_Q4_01). **B**) The highest scoring motif discovered in low c-Fos peaks in K562 is similar to the canonical motif. **C**) The 23rd highest scoring motif in high c-Fos peaks in K562 has the best resemble to the canonical motif, but is still quite different from the motif depicted in A). Click here for file

Additional file 6: Figure S5 After balancing GC-content, higher peaks in K562 still have more consistent support in other data marking regulatory regions. As Figure 3, but for a GC-balanced subset of K562 peaks. For each TF, peaks were binned into 10 equal-interval bins based on GC-content after removing the top and bottom 5% (GC-outliers). Then, for each height bin, we randomly sampled the same number of peaks from each GC-bin to keep GC-content approximately equal in each height bin. The trend in the data is similar to the trend in Figure 3. Click here for file

Additional file 7: Figure S6 Low cell-type specific peaks have differences in chromatin accessibility. As Figure 4, but for the 30% lowest peaks. The 30% lowest peaks show clear differences in chromatin accessibility. Click here for file

Additional file 8: Figure S7 Low cell-type specific peaks have differences in active histone modifications. As Figure 5, but for the 30% lowest peaks. The 30% lowest peaks show clear differences in active histone markings. Click here for file

Additional file 9: Figure S8 High cell-type specific peaks have a larger difference in chromatin signal than low peaks. **A**) Peaks were binned in equally-sized bins sorted on peak height. The upper panel (K562) shows the difference in median DNase-seq read count pr bin between K562 and HeLa-S3 for K562 specific peaks, whereas the lower panel shows the difference between HeLa-S3 and K562 for HeLa-S3 specific peaks. **B**) As A), but for H3K4me3 signal instead of DNase accessibility. Within the set of peaks that are unique to a cell type, the higher peaks have larger difference in chromatin accessibility and active marks between the cell types than lower peaks. This suggests that higher cell-type specific peaks are more likely to be due to cell-type specific regulation of the chromatin. Click here for file

Additional file 10: Figure S9 ROC curves. **A**) ROC curves on 10-fold stratified cross-validation on K562 and HeLa-S3 training and testing data. X-axis is true positive rate, Y-axis is 1-false positive rate. **B**) ROC curves on 10-fold stratified cross-validation on K562/HeLa-S3 training and GM12878 testing data. Click here for file

Additional file 11: Figure S10 Most important feature group for classification. Difference in ROC score after first removing confounding factors (peak height and clustering), and grouping features into three groups (cell-type specific, promoter/sequence, phyloP) and then removing a given feature group. Error bars show average ROC score change on 10 cross-validation folds plus/minus one standard deviation. Click here for file

Additional file 12: Figure S11 K562 peaks overlap. Overlap in different cell types for all K562 peaks. CTCF and GABP have many common peaks between all the cell types, whereas c-Fos have few common peaks between all cell types. Click here for file

Additional file 13: Table S1 Significant co-factors. This table shows the co-factors having significant expression differences between K562 and HeLa-S3. Co-factors shown in bold text also had a PWM available and significant difference in PWM score distributions between cell-type specific peaks. Click here for file

Additional file 14: Table S2 PWMs. The position weight-matrix identifiers of the PWMs taken from Transfac Professional [57] (6) and Jaspar [58] (1) databases. PWMs were not available for the TFs BDP1, BRF1, BRF2, RPC155, TAF1, and TFIIIC-110. If more than one PWM was available for a given TF, the PWM with the highest information content after division by PWM length was chosen. Also shown are the sequence logos made from the motif sequences using WebLogo [68]. Click here for file
