# Supplementary figures and images for: Cell-type specificity of ChIP-predicted transcription factor binding sites
Source: BMC Genomics. 2012 Aug 3;13:372. doi: 10.1186/1471-2164-13-372 (PMC3574057; doi:10.1186/1471-2164-13-372)

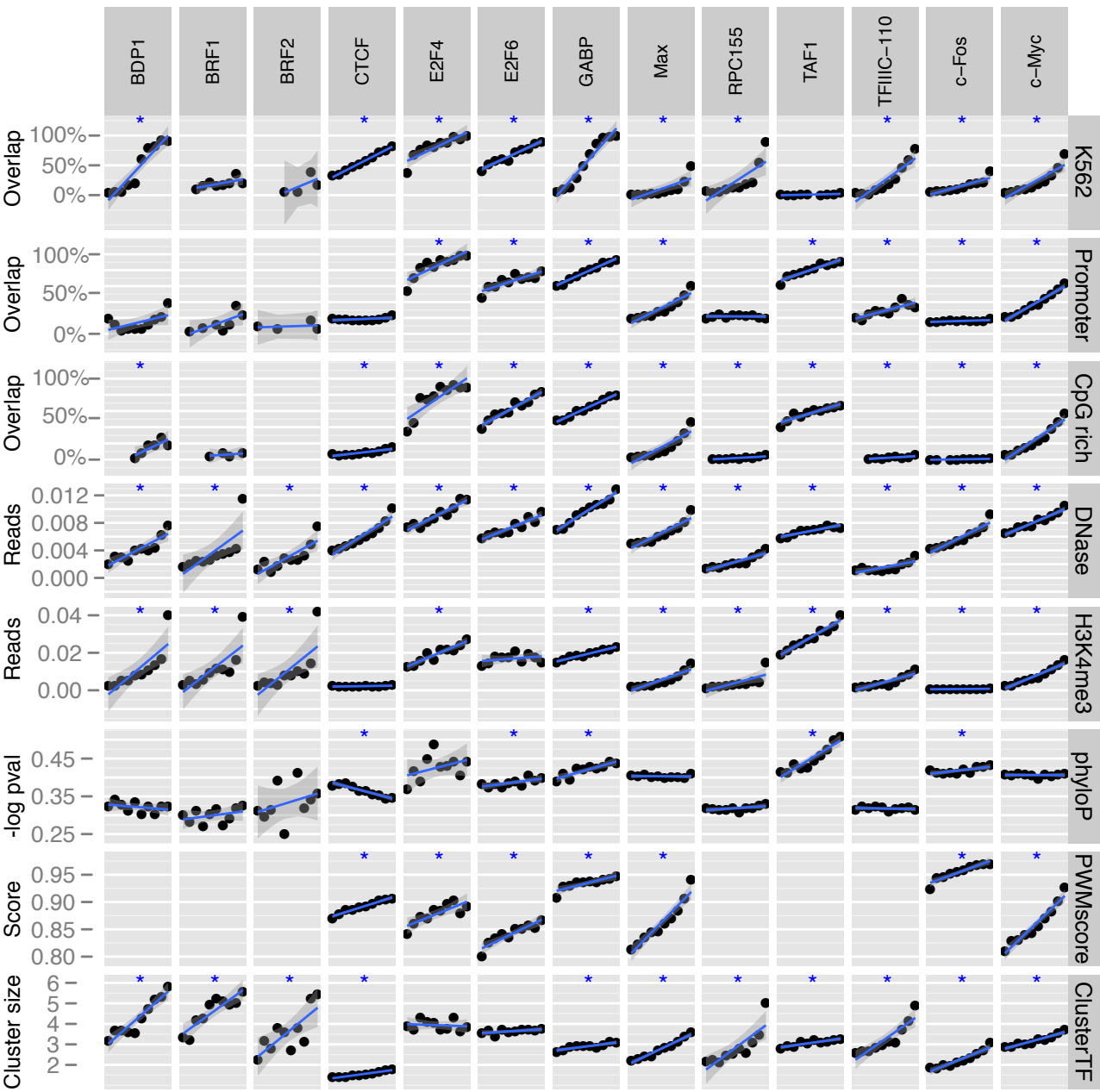

Supplement: Additional file 2: Figure S1 — Higher peaks in HeLa-S3 have more consistent support in other data marking regulatory regions. As Figure 3, but for HeLa-S3 peaks instead of K562. Overlap is here measured as percentage of HeLa-S3 peaks that overlap with K562 peaks. [file 1471-2164-13-372-S2.pdf]

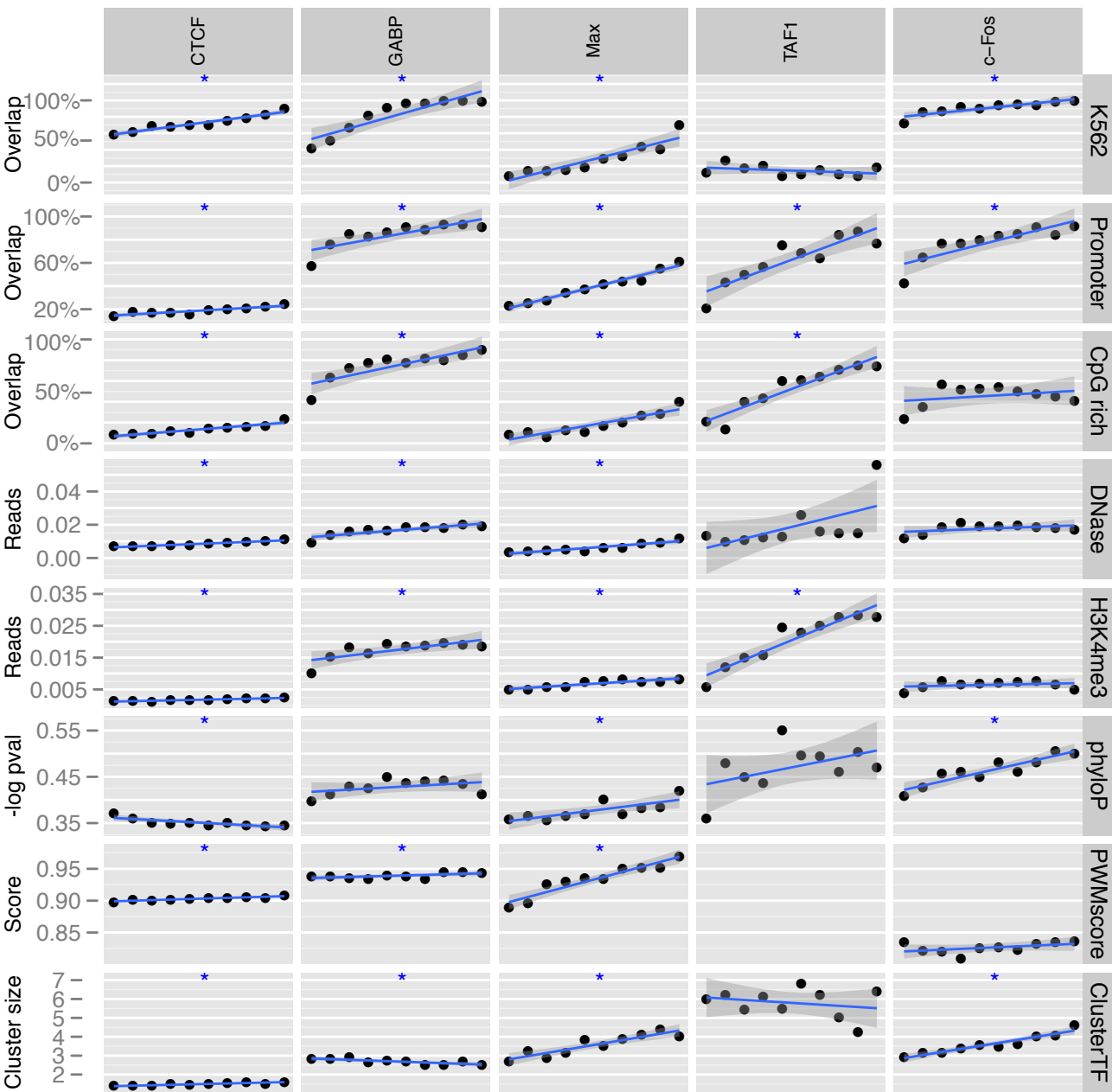

Supplement: Additional file 3: Figure S2 — Higher peaks in GM12878 have more consistent support in other data marking regulatory regions. As Figure 3, but for GM12878 peaks. Overlap is here measured as percentage of GM12878 peaks that overlap with K562 peaks. [file 1471-2164-13-372-S3.pdf]

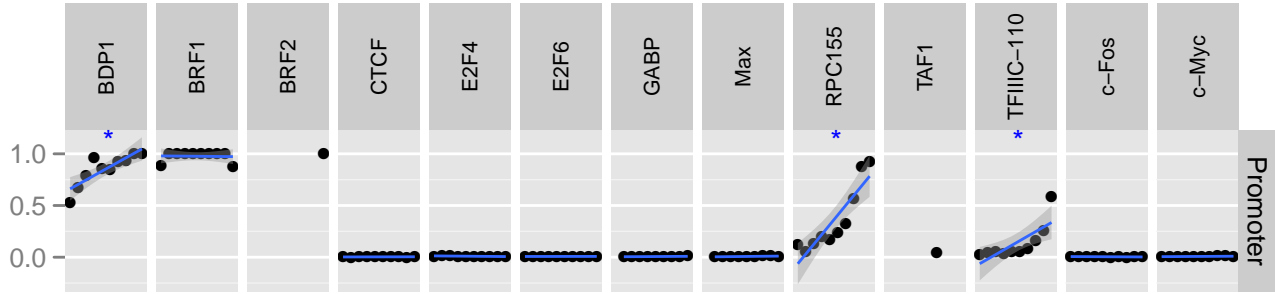

Supplement: Additional file 4: Figure S3 — Pol III promoter overlap correlates with peak height for Pol III-associated factors. Similar to Promoter data in Figure 3, but for overlap with Pol III promoters, (defined as the regions -2000bp upstream and +200bp downstream of tRNA transcription start sites from the tRNAscan-SE Genomic tRNA Database [56]). The Pol III-associated factors and subunits BDP1, RPC155 and TFIIIC-110 all show significant correlation between peak height and promoter overlap. [file 1471-2164-13-372-S4.pdf]

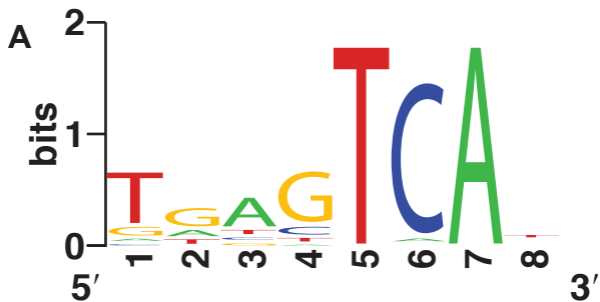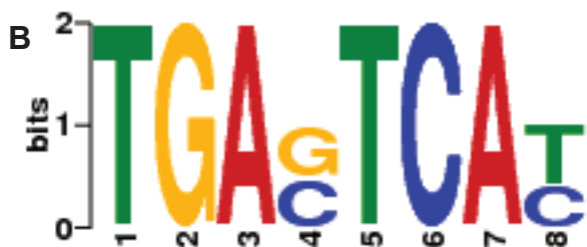

DFENE 01.12.2011 13:45

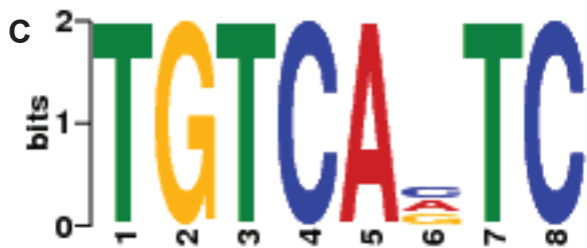

DFENE 01.12.2011 13:26

Supplement: Additional file 5: Figure S4 — Different motifs in low and high c-Fos peaks. A) The canonical AP-1 motif taken from the Transfac database [57] (matrix identifier V$AP1_Q4_01). B) The highest scoring motif discovered in low c-Fos peaks in K562 is similar to the canonical motif. C) The 23rd highest scoring motif in high c-Fos peaks in K562 has the best resemble to the canonical motif, but is still quite different from the motif depicted in A). [file 1471-2164-13-372-S5.pdf]

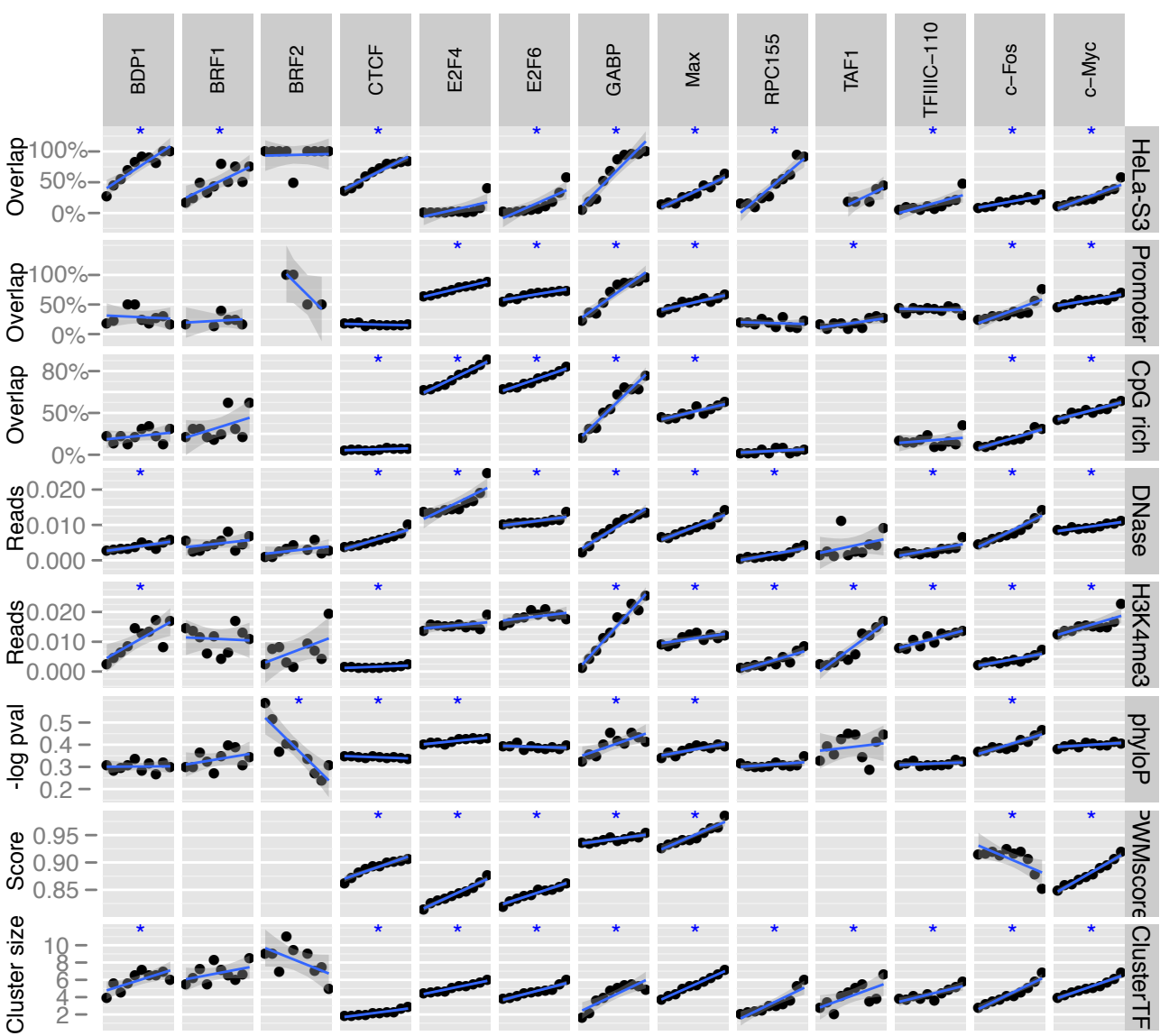

Supplement: Additional file 6: Figure S5 — After balancing GC-content, higher peaks in K562 still have more consistent support in other data marking regulatory regions. As Figure 3, but for a GC-balanced subset of K562 peaks. For each TF, peaks were binned into 10 equal-interval bins based on GC-content after removing the top and bottom 5% (GC-outliers). Then, for each height bin, we randomly sampled the same number of peaks from each GC-bin to keep GC-content approximately equal in each height bin. The trend in the data is similar to the trend in Figure 3. [file 1471-2164-13-372-S6.pdf]

DNase reads per mill.

**A**

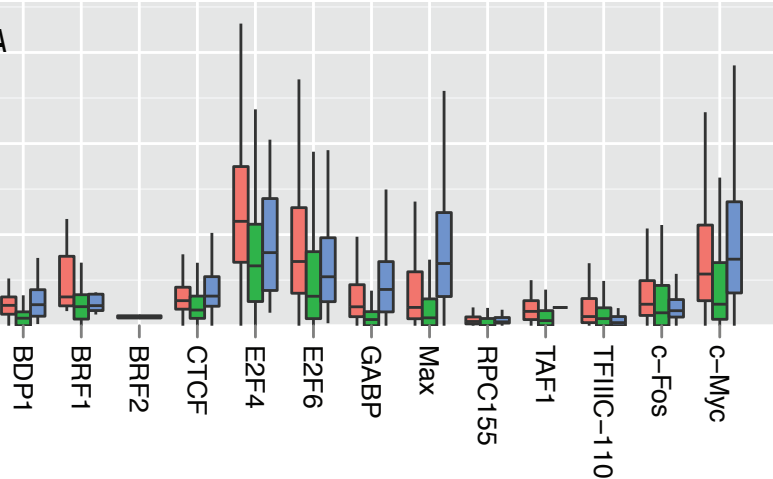

**DNase (K562 peaks)**

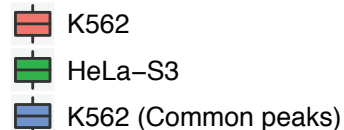

DNase reads per mill.

**B**

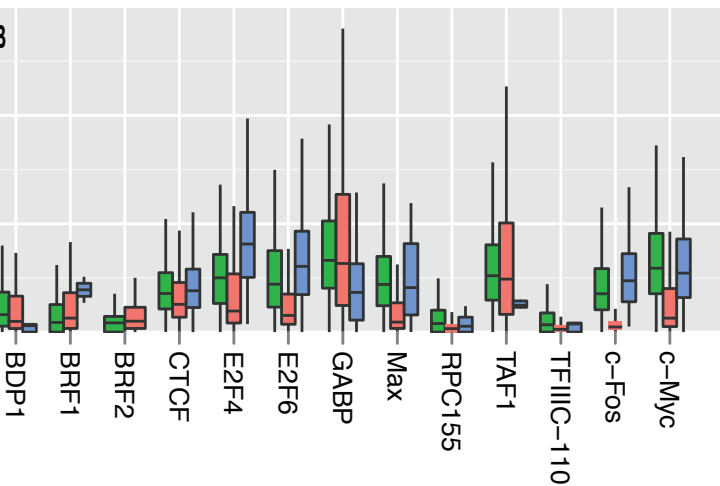

**DNase (HeLa-S3 peaks)**

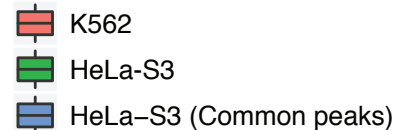

Supplement: Additional file 7: Figure S6 — Low cell-type specific peaks have differences in chromatin accessibility. As Figure 4, but for the 30% lowest peaks. The 30% lowest peaks show clear differences in chromatin accessibility. [file 1471-2164-13-372-S7.pdf]

H3K4me3 reads per mill.

**A**

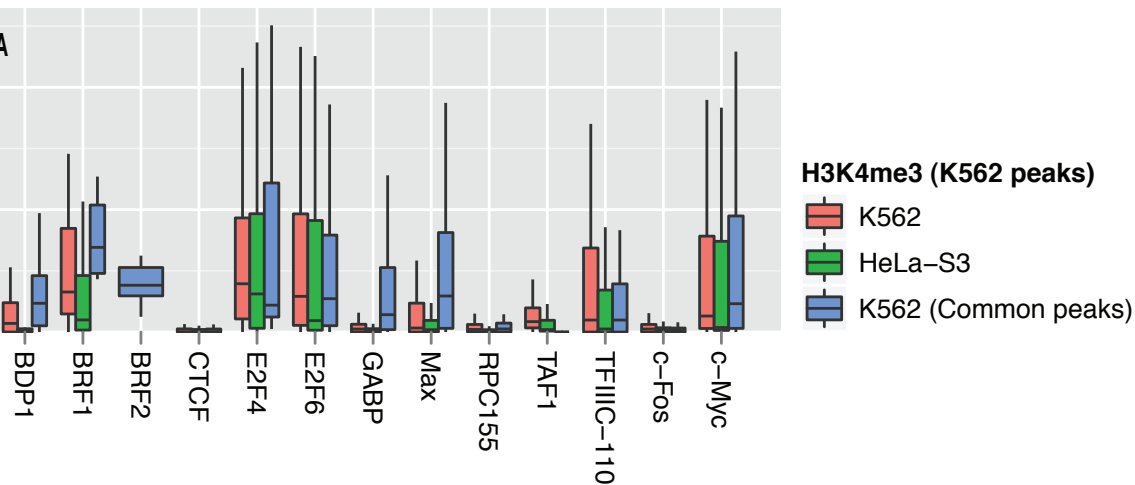

H3K4me3 reads per mill.

**B**

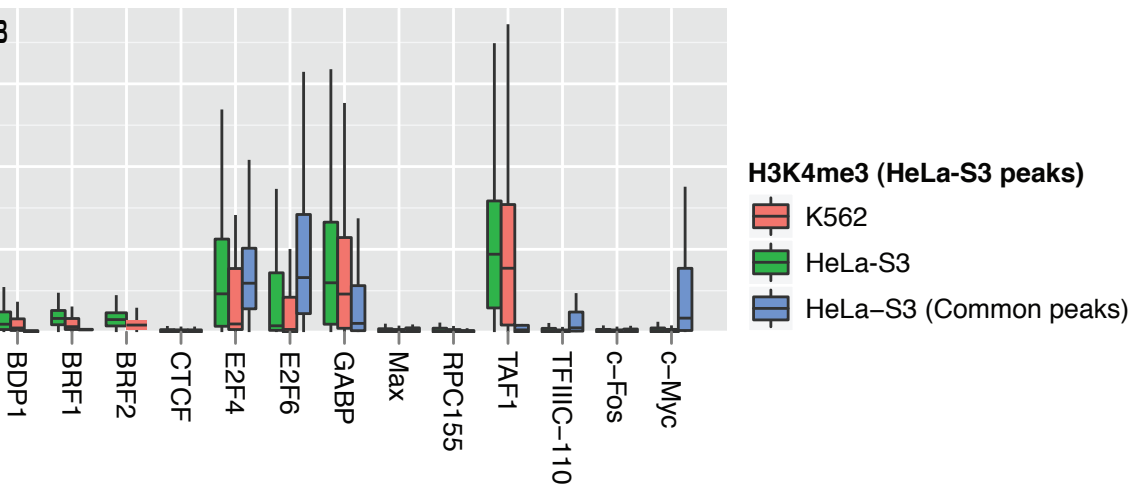

Supplement: Additional file 8: Figure S7 — Low cell-type specific peaks have differences in active histone modifications. As Figure 5, but for the 30% lowest peaks. The 30% lowest peaks show clear differences in active histone markings. [file 1471-2164-13-372-S8.pdf]

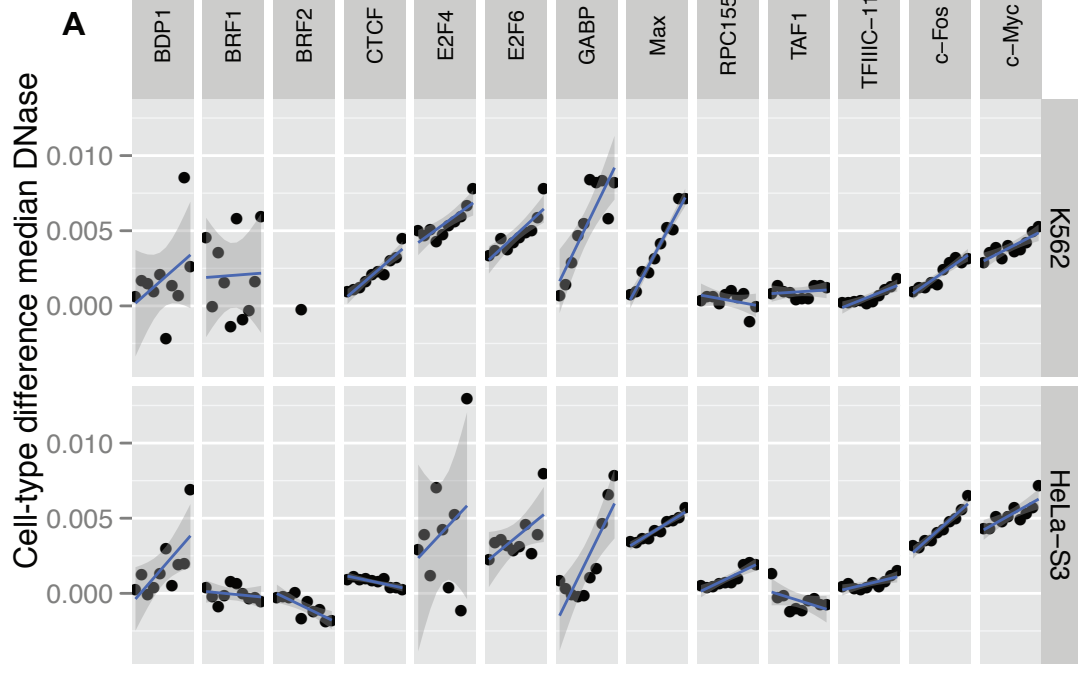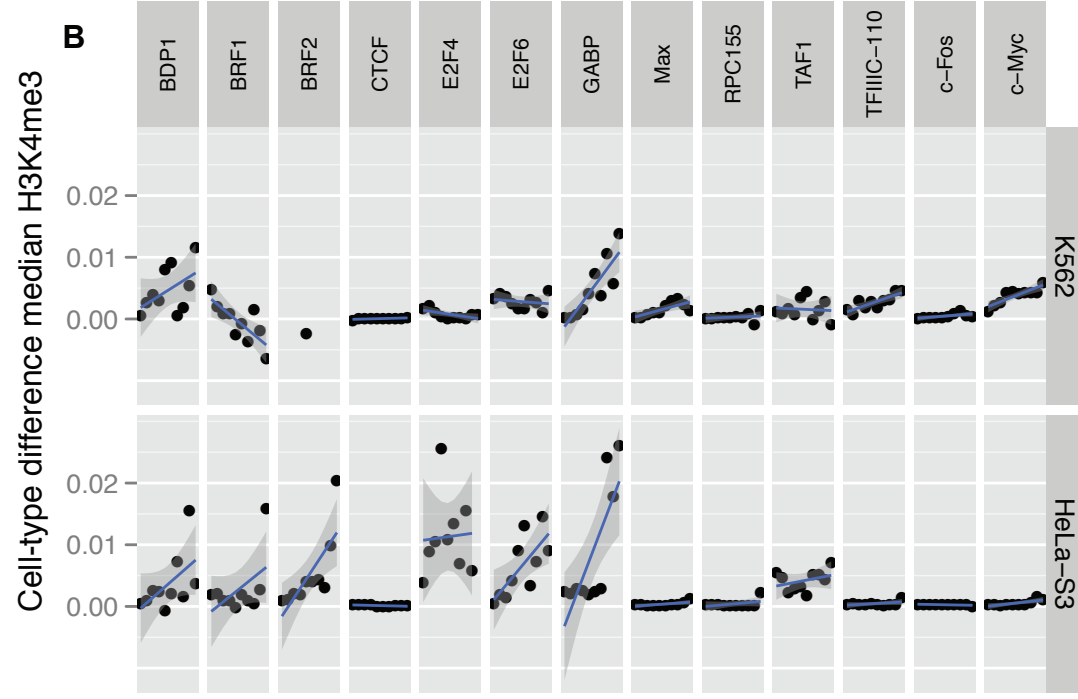

Supplement: Additional file 9: Figure S8 — High cell-type specific peaks have a larger difference in chromatin signal than low peaks. A) Peaks were binned in equally-sized bins sorted on peak height. The upper panel (K562) shows the difference in median DNase-seq read count pr bin between K562 and HeLa-S3 for K562 specific peaks, whereas the lower panel shows the difference between HeLa-S3 and K562 for HeLa-S3 specific peaks. B) As A), but for H3K4me3 signal instead of DNase accessibility. Within the set of peaks that are unique to a cell type, the higher peaks have larger difference in chromatin accessibility and active marks between the cell types than lower peaks. This suggests that higher cell-type specific peaks are more likely to be due to cell-type specific regulation of the chromatin. [file 1471-2164-13-372-S9.pdf]

**A**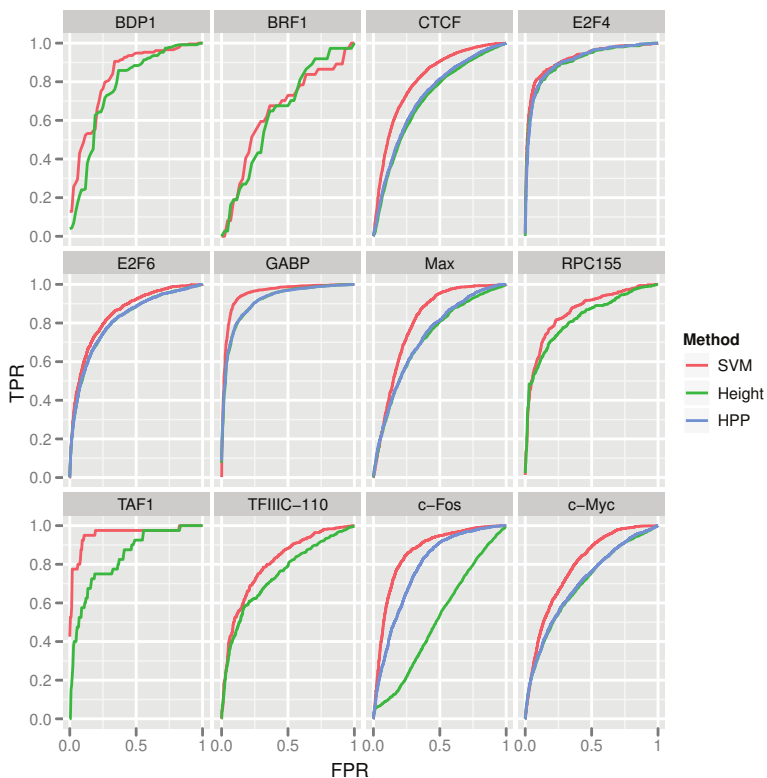**B**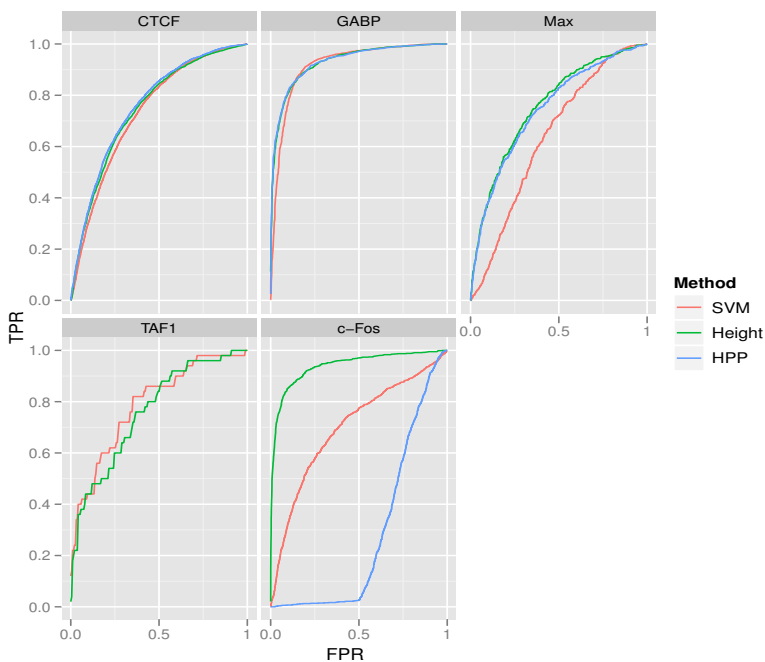

Supplement: Additional file 10: Figure S9 — ROC curves. A) ROC curves on 10-fold stratified cross-validation on K562 and HeLa-S3 training and testing data. X-axis is true positive rate, Y-axis is 1-false positive rate. B) ROC curves on 10-fold stratified cross-validation on K562/HeLa-S3 training and GM12878 testing data. [file 1471-2164-13-372-S10.pdf]

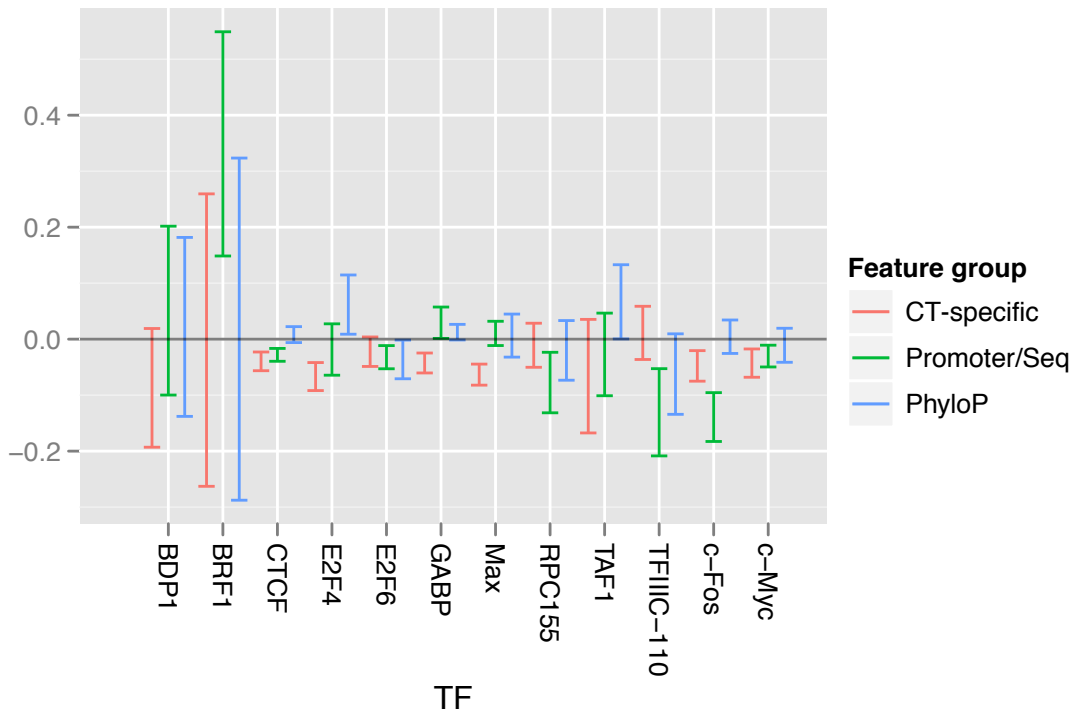

Supplement: Additional file 11: Figure S10 — Most important feature group for classification. Difference in ROC score after first removing confounding factors (peak height and clustering), and grouping features into three groups (cell-type specific, promoter/sequence, phyloP) and then removing a given feature group. Error bars show average ROC score change on 10 cross-validation folds plus/minus one standard deviation. [file 1471-2164-13-372-S11.pdf]

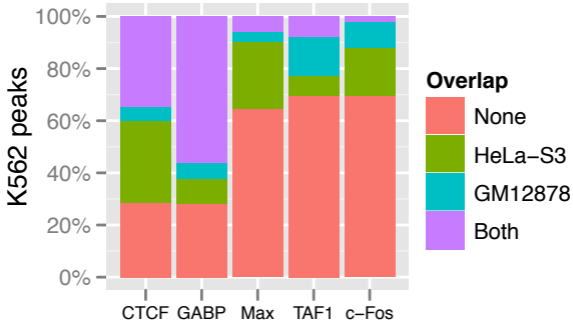

Supplement: Additional file 12: Figure S11 — K562 peaks overlap. Overlap in different cell types for all K562 peaks. CTCF and GABP have many common peaks between all the cell types, whereas c-Fos have few common peaks between all cell types. [file 1471-2164-13-372-S12.pdf]
