# Supplementary material for: Cell-type specificity of ChIP-predicted transcription factor binding sites
Source: BMC Genomics. 2012 Aug 3;13:372. doi: 10.1186/1471-2164-13-372 (PMC3574057; doi:10.1186/1471-2164-13-372)
Supplement: Additional file 13: Table S1 — Significant co-factors. This table shows the co-factors having significant expression differences between K562 and HeLa-S3. Co-factors shown in bold text also had a PWM available and significant difference in PWM score distributions between cell-type specific peaks. [file 1471-2164-13-372-S13.pdf]

Cell-type Specificity of ChIP-predicted Transcription Factor Binding Sites

Tony Håndstad[1], Morten Beck Rye[1], Rok Močnik[1], Finn Drabløs[1], Pål Sætrom[1,2,*]
1 Department of Cancer Research and Molecular Medicine, Norwegian University of Science and Technology, NO-7491, Trondheim, Norway
2 Department of Computer and Information Science, Norwegian University of Science and Technology, NO-7491, Trondheim, Norway
∗ E-mail: pal.satrom@ntnu.no

Supplementary Table S1 - Significant co-factors

This table shows the co-factors having significant expression differences between K562 and HeLa-S3. Co-factors shown in bold text also had a PWM available and significant difference in PWM score distributions between cell-type specific peaks.

TF	Significant co-factors
BDP1	
BRF1	**TP53**, GTF3C5
BRF2	
CTCF	
E2F4	HDAC1
E2F6	BMI1
GABP	**LAP3**
Max	**MYC, MXI1,** TIPARP
RPC155	
TAF1	**TP53, FOS**
TFIIIC-110	
c-Fos	**JUNB, LAP3, NFATC3,** BCL3, TSC22D3
c-Myc	**MXI1**, ELF3
