# Supplementary material for: Cell-type specificity of ChIP-predicted transcription factor binding sites
Source: BMC Genomics. 2012 Aug 3;13:372. doi: 10.1186/1471-2164-13-372 (PMC3574057; doi:10.1186/1471-2164-13-372)
Supplement: Additional file 14: Table S2 — PWMs. The position weight-matrix identifiers of the PWMs taken from Transfac Professional [57] (6) and Jaspar [58] (1) databases. PWMs were not available for the TFs BDP1, BRF1, BRF2, RPC155, TAF1, and TFIIIC-110. If more than one PWM was available for a given TF, the PWM with the highest information content after division by PWM length was chosen. Also shown are the sequence logos made from the motif sequences using WebLogo [68]. [file 1471-2164-13-372-S14.pdf]

Cell-type Specificity of ChIP-predicted Transcription Factor Binding Sites

Tony Håndstad[1], Morten Beck Rye[1], Rok Močnik[1], Finn Drabløs[1], Pål Sætrom[1,2,*]
1 Department of Cancer Research and Molecular Medicine, Norwegian University of Science and Technology, NO-7491, Trondheim, Norway
2 Department of Computer and Information Science, Norwegian University of Science and Technology, NO-7491, Trondheim, Norway
∗ E-mail: pal.satrom@ntnu.no

Supplementary Table S2 - PWMs

The position weight-matrix identifiers of the PWMs taken from Transfac Professional [1] (6) and Jaspar [2] (1) databases. PWMs were not available for the TFs BDP1, BRF1, BRF2, RPC155, TAF1, and TFIIIC-110. If more than one PWM was available for a given TF, the PWM with the highest information content after division by PWM length was chosen. Also shown are the sequence logos made from the motif sequences using WebLogo [3].

References

1. Matys V, Fricke E, Geffers R, Gossling E, Haubrock M, et al. (2003) TRANSFAC: Transcriptional regulation, from patterns to profiles. Nucleic Acids Research 31: 374.

2. Bryne J, Valen E, Tang M, Marstrand T, Winther O, et al. (2008) Jaspar, the open access database of transcription factor-binding profiles: new content and tools in the 2008 update. Nucleic Acids Research 36: D102–D106.

3. Crooks G, Hon G, Chandonia J, Brenner S (2004) Weblogo: a sequence logo generator. Genome research 14: 1188–1190.

TF	PWM	Logo
CTCF	MA0139.1	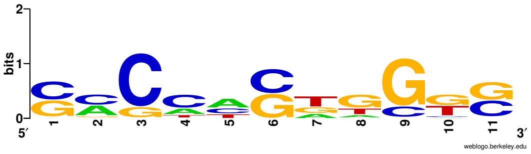
E2F4	V$E2F1_Q4_01	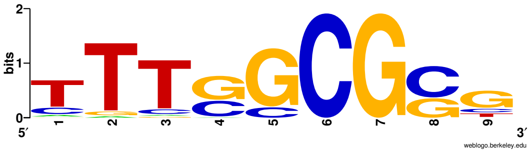
E2F6	V$E2F1_Q4_01	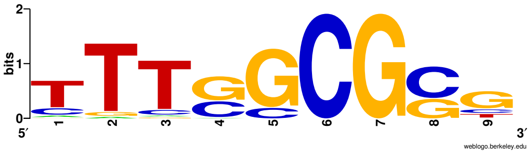
GABP	V$ETS_Q6	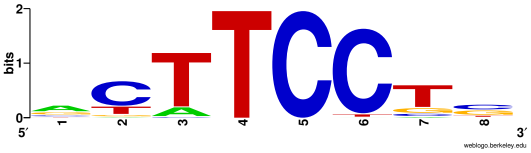
Max	V$MYC_Q2	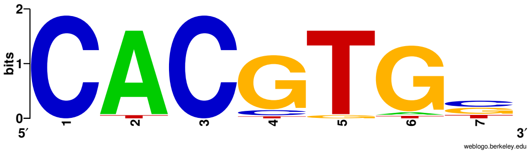
c-Fos	V$AP1_Q4_01	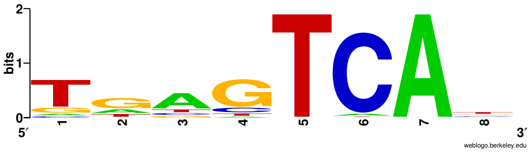
c-Myc	V$MYC_Q2	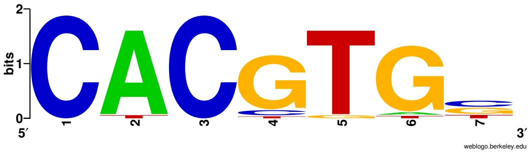
